# Supplementary material for: Developing a multimedia patient-reported outcomes measure for low literacy patients with a human-centered design approach
Source: PLoS One. 2024 Jun 5;19(6):e0304351. doi: 10.1371/journal.pone.0304351 (PMC11152264; doi:10.1371/journal.pone.0304351)
Supplement: S1 Dataset — (DOCX) [file pone.0304351.s001.docx]

Minimal Data Set

PONE-D-23-24670 - Developing a multimedia patient-reported outcomes measure for low literacy patients

Table of Contents

[OBSERVATION DATA 2](#_Toc153910747)

[Multimedia PROM Project Direct Observation Data Collection Form - 1 2](#_Toc153910748)

[Multimedia PROM Project Direct Observation Data Collection Form - 2 5](#_Toc153910749)

[Multimedia PROM Project Direct Observation Data Collection Form - 3 8](#_Toc153910750)

[Multimedia PROM Project Direct Observation Data Collection Form - 4 12](#_Toc153910751)

[Multimedia PROM Project Direct Observation Data Collection Form - 5 15](#_Toc153910752)

[Multimedia PROM Project Direct Observation Data Collection Form - 6 18](#_Toc153910753)

[DISCOVERY INTERVIEW GUIDES 21](#_Toc153910754)

[Target Participant: Patients 21](#_Toc153910755)

[Target Participant: Caregivers 24](#_Toc153910756)

[Target Participant: Clinic Staff 26](#_Toc153910757)

[IDEATION WORKSHOP OUTLINE 28](#_Toc153910758)

[Ideation Worksheet 30](#_Toc153910759)

[Ideation Worksheet Example 31](#_Toc153910760)

[MEMBER CHECKING INTERVIEW GUIDE 32](#_Toc153910761)

# OBSERVATION DATA

## Multimedia PROM Project Direct Observation Data Collection Form - 1

Clinic: Surgeon and therapy clinic / fellow side

Location in clinic: CNHC clinic waiting area / check-in counter

Date: 11/2/20

Day of week: Monday

The main goals of this data collection are to:

1. Understand the process/flow, routine, and environment of clinic, specifically as it relates to completion of intake surveys and PROMs via the OBERD system

2. Observe scenarios in which patients can or cannot complete intake surveys and PROMs

3. Observe scenarios in which clinic staff need to assist or encourage patients to complete intake surveys and PROMs

4. Use data to inform the PAB meeting and to update the discovery interview guide

5. Use data to generate graphics for use in PAB and future interviews

| **Prompt/ Category** | **What was said?** | **What did you see? What happened? Perception of emotions?** |
| --- | --- | --- |
| Steps of OBERD completion from patient perspective |  |  |
| Patient discussion of OBERD |  |  |
| Staff discussion of OBERD |  |  |
| Patient/ staff interactions surrounding OBERD | 8:22: “Thank you for doing your OBERD”    8:23: another patient, “Have you done your OBERD”; “This is my second visit, they told me to come on Monday.”; “Yes I understand but you need to fill out his form every time you come”  9:03: “did you fill out your registration forms online?” “no, I don’t think I got them.” “yeah, sometimes it goes to spam. I’ll give you one now.”  9:05: “Did you do your online registration?” “No I did not.” “Ok, I’ll give you the forms now” | Still given a piece of paper to fill out on a clipboard.  8:23: The patient is left-handed and his injury on his left thumb (where he is wearing a splint). He is struggling to hold the pen while filling out the paper form. He takes out his wallet and takes out a piece of paper. He unfolds piece of paper. It is a lined piece of notebook paper with handwriting on it. He is referencing it as he is filling out the form. He is holding the clipboard in his lap. Folds the piece of paper back up and puts it back into his wallet. Flips the form around to back. Seems that he is deliberately filling out the form, taking time to read the questions and select an answer (i.e., not just going down and selecting whatever answer.) Seems to have to reread some things a few times. As he is reading, using pen to point to what he is reading. Can see him deliberating between answer choices. Can tell that he is taking the form seriously and trying to do his best to answer.  8:31: patient is done completing the form, turns it back in  She sits down, also left-handed, is wearing a splint on her left hand/wrist. Filling out the forms. Pulls out a stack of printed papers. Looking through the many pages that she has. Seems to have found what she is looking for. As she maneuvers the forms, she puts the stack of papers under her right arm, holding it there. Doesn’t seem to be having any trouble filling out the form with her left hand despite having the splint on.  9:13: hands the forms back in  9:07: patient finishes form very quickly and turns it back in. He is then immediately called back for his appointment. |
| Easy patient interactions with OBERD device |  | 8:50: patient sits down with his clipboard. He starts to fill it out. He’s wearing gloves (seems to be a COVID precaution). Doesn’t have any upper extremity injuries as far as I can tell. Takes out his phone from pant pocket and starts looking at something.  8:56: puts his phone back in pocket, starts working on the form again. Not sure what he was doing on his phone (whether related to filling out the form or not). Turns the first page over, starts filling out the second page. (Sometimes the forms are front and back, other time it seems that they’re not front and back.)  8:58: flipped onto 3^rd^ page  8:59: now on 4^th^ page, doesn’t seem to be having very much trouble, he is now cruising  9:02: flips over to the last page, this seems to be the actual PROs sheet.  9:05: turns the form back in |
| Difficult patient interactions with OBERD device |  |  |
| Easy staff interactions with OBERD device |  |  |
| Difficult staff interactions with OBERD device |  |  |
| Other |  | None of the patients had caregivers with them today; they all came to their appointments by themselves |

## Multimedia PROM Project Direct Observation Data Collection Form - 2

Clinic: Surgeon and therapy clinic / fellow clinic

Location in clinic: CNHC clinic waiting area / check-in counter

Date: 10/20/20

Day of week: Tuesday

The main goals of this data collection are to:

1. Understand the process/flow, routine, and environment of clinic, specifically as it relates to completion of intake surveys and PROMs via the OBERD system

2. Observe scenarios in which patients can or cannot complete intake surveys and PROMs

3. Observe scenarios in which clinic staff need to assist or encourage patients to complete intake surveys and PROMs

4. Use data to inform the PAB meeting and to update the discovery interview guide

5. Use data to generate graphics for use in PAB and future interviews

| **Prompt/ Category** | **What was said?** | **What did you see? What happened? Perception of emotions?** |
| --- | --- | --- |
| Steps of OBERD completion from patient perspective |  |  |
| Patient discussion of OBERD |  |  |
| Staff discussion of OBERD |  |  |
| Patient/ staff interactions surrounding OBERD | 1:15pm: “I see you started the questionnaire but you didn’t finish it, I see you did one question but you have about 99 left to go, you see there is only one green one. Want me to log you onto one of these computers [iPads] so you can fill it out?”; “This is the one question you filled out right? You are 6 feet tall? You filled that out?”; “The screen will move by itself…”  “Can I ask you some questions about your health?” “You can really move a chair with that hand?” “Last little bit…” “You can’t hold a frying pan in your hand, right?” “So is your pain very mild?” “Do you want me to put neither agree nor disagree?” “Although I bother you all the time, you still like me?”  “I did the online thing already”  “Thank you for doing that. That saved you a lot of time, didn’t it”  “You hit start, and it’ll take you to the next screen.” “If nothing applies, just put a big check box that says none”  “Your follow-up questionnaire wasn’t done Ms. Janet.” “Start here, you need to fill out both front and back”  “Thank you for doing your OBERD at home. And it’s long, isn’t it. That is why I tell patients they should fill it out at home.”  “You can stand right here and do this. Here is a pen.” | This patient is younger, in 20s or 30s. Appears bored. Willing to fill out the questionnaire; doesn’t put up a fight; says he will do it. Paula wipes down the iPad, pulls up the survey, shows him how to do it, gives him the iPad. So in this instance the patient fills it out themselves. Since he had to fill out info about his height, seems that he is a new patient.  1:20pm: Pt walks away with iPad  1:33pm: Pt returns with iPad  Dorothy asks patient all the questions for this post-op patient at the check in counter. Patient willingly answers all the questions. When asking the questions, they become yes/no questions (she doesn’t read all the responses). There are some leading questions.  Pt said this before anyone even prompted him  This is a more elderly gentleman. Given iPad at 1:53 pm. Comes back with iPad at 1:59pm. No discussion of whether he had any difficulty completing it.  2:01pm: She is given a paper form and a pen.  2:02pm: She gives form back.  This patient got the paper form because she came in late for her appointment, and she isn’t Dr. Giladi’s patient. She is Dr. Baugher’s patient. Dr. Giladi wants OBERD filled out even when the patients are late. For Dr. Baugher’s patients, if they are late, he doesn’t want them held up at the front desk, he wants them to be sent back for appointment. So they fill out the paper form, which is a distilled version of what’s on OBERD, so takes much less time to complete. This is then scanned into the patient chart, and both patients and providers can see.  Another patient who is filling the form out by hand. |
| Easy patient interactions with OBERD device |  | Patients don’t appear to be having trouble with filling out OBERD. None have complained or mentioned any difficulty with completing the surveys |
| Difficult patient interactions with OBERD device |  |  |
| Easy staff interactions with OBERD device |  | Dorothy goes through the next day’s patients, sees who has or hasn’t filled out their surveys. For those that haven’t, she clicks “remind” button, which sends them an email reminder. She does this 3x a day. It speeds up the check-in process a lot when they don’t have to fill it out in the clinic |
| Difficult staff interactions with OBERD device |  |  |
| Other |  | 2 check-in ladies; 2 translator phones with various interpretation services provided; cabinet of iPads that are available for patients to fill out; |

Debriefing notes:

- One patient asked what I was doing there
- Per Dorothy, was not a busy day, this was considered a light day, the other clinic was open as well
- No more patients to be seen by 3pm on this day

Schematic of clinic space:

Complete account of the observation:

Summary/write-up including interpretations, reflections, and insights:

- Insight: self-administration is better than being administered by someone else!
  - Because when administered by front desk ladies, they don’t offer all the answers options (takes too long!), ask leading questions, start assuming answers for certain questions once they’re a few in, or make assumptions based on what they see the patient’s injury is; certainly seems that there is potential for introducing bias
- Overall, for patients who haven’t filled out their OBERD before their appointment, there are 3 ways that it can go:
  - 1) Patient filled it out on iPad
  - 2) Front desk ladies help them fill it out
  - 3) For patients who are also late and aren’t Dr. Giladi’s patients, they fill out an abbreviated paper form
- Need to figure out: why does the front desk sometimes prefer to fill it out for the patient? (Especially since this takes a lot of extra time to do for the staff). Is it because patients struggle with the iPad?

## Multimedia PROM Project Direct Observation Data Collection Form - 3

Clinic: Surgeon and therapy clinic / fellow clinic

Location in clinic: CNHC clinic waiting area / check-in counter

Date: 10/21/20

Day of week: Wednesday

The main goals of this data collection are to:

1. Understand the process/flow, routine, and environment of clinic, specifically as it relates to completion of intake surveys and PROMs via the OBERD system

2. Observe scenarios in which patients can or cannot complete intake surveys and PROMs

3. Observe scenarios in which clinic staff need to assist or encourage patients to complete intake surveys and PROMs

4. Use data to inform the PAB meeting and to update the discovery interview guide

5. Use data to generate graphics for use in PAB and future interviews

| **Prompt/ Category** | **What was said?** | **What did you see? What happened? Perception of emotions?** |
| --- | --- | --- |
| Steps of OBERD completion from patient perspective |  | Patient goes to check-in counter -> handed a wiped down iPad by front desk ladies -> given brief instructions on how to use the iPad -> they sit back down in waiting room to complete the survey on the iPad |
| Patient discussion of OBERD |  |  |
| Staff discussion of OBERD |  |  |
| Patient/ staff interactions surrounding OBERD |  |  |
| Easy patient interactions with OBERD device |  | 9:35am, patient walks from check-in counter back to waiting room with her device (but sat somewhere that I don’t have a direct view)  9:42am: same patient returns the iPad  This is an older woman, maybe in 60s or so  9:56am: another middle-aged woman walks out with iPad. She is using both hands to complete it. She takes out a small piece of paper from her purse and looks at it; appears that she is referencing the information on that sheet of paper to fill out the form. Has to balance the iPad on the armchair, while holding this piece of paper. She puts this piece of paper away and then gets another piece of paper. Looks like first piece of paper was printed, and 2^nd^ is hand-written. Opens and closes and examines this paper several times.  10:10am: patient returns to counter with iPad. Has a calm encounter  10:12am: patient sits down again with the iPad. It appears that maybe she had a question, or it was still not fully completed (unable to hear conversation with her and front desk lady). She is now holding iPad with one hand while completing with the other hand. Seems that she is moving through the instrument relatively slowly.  10:21am: Patient gets back in line while holding the iPad  10:23am: she makes it to the front of the line, gives the iPad back.  10:26am: she sits back down, and continues to wait for her appointment  10:30am: a middle-aged gentleman sits down with iPad. iPad is balanced on his right leg. He does not appear to be having trouble navigating it. He is using the keyboard to type things.  10:35am: Reaches into his pant pocket and takes out his cell phone, is now making a call (or potentially picked up a call) and speaking to someone.  10:36am: Repeatedly swiping up and down on the iPad rather quickly, the severity of his motions makes him seem mildly annoyed, seems to be looking for the form.  10:40am: hangs up the phone. The iPad screen has gone dark. Goes to front desk. Comes back with iPad still in hand. iPad sitting on his right leg and he is looking at his phone. Phone is in one hand and iPad is on leg; he is looking between the two devices; seems to be finding information on his phone. Sometimes typing on iPad keyboard on screen, sometimes clicking point-and-click buttons. Also has a paper in his lap that he consulted.  10:50am: patient is back in line with iPad in hand. There is one person in front of him  10:51am: sat back down again. Not sure why he stood up. Looks like he is completing questions again.  10:52am: back at counter, and handed iPad back in. Sits back down |
| Difficult patient interactions with OBERD device |  |  |
| Easy staff interactions with OBERD device |  |  |
| Difficult staff interactions with OBERD device |  |  |
| Other |  | Several patients sitting in waiting room on their phones, some not on their phones.  Many patients in waiting room have splints on, which makes it more difficult than the typical patient population for them to hold devices (including their own phones); they need to be able to complete the surveys with just one working/functioning hand  10:37am: 5 of 6 patients in waiting room are currently on their phones. |

Debriefing notes:

- There are many lulls, in terms of data-rich collection periods. Sometimes patients are just waiting in the waiting area, and I do not see

Summary/write-up including interpretations, reflections, and insights:

- Problem: some patients had difficulty maneuvering the iPad—for ex, as they were balancing it on their leg. This is especially problematic for hand/upper extremity patients.
  - Insight: Need to create something that patients don’t need to hold. Mount the mPROM on some sort of stand? Incorporate it into some sort of a station or booth?
- It can take a long time, but this is in part due to all the other PROMs (in addition to PROMIS UE) and the intake survey that they must fill out. Patients not only were seen to get annoyed, but this can delay their appointment.
  - Insight: Will be important to not increase the amount of time it takes to complete the mPROM (as opposed to the text-based one)


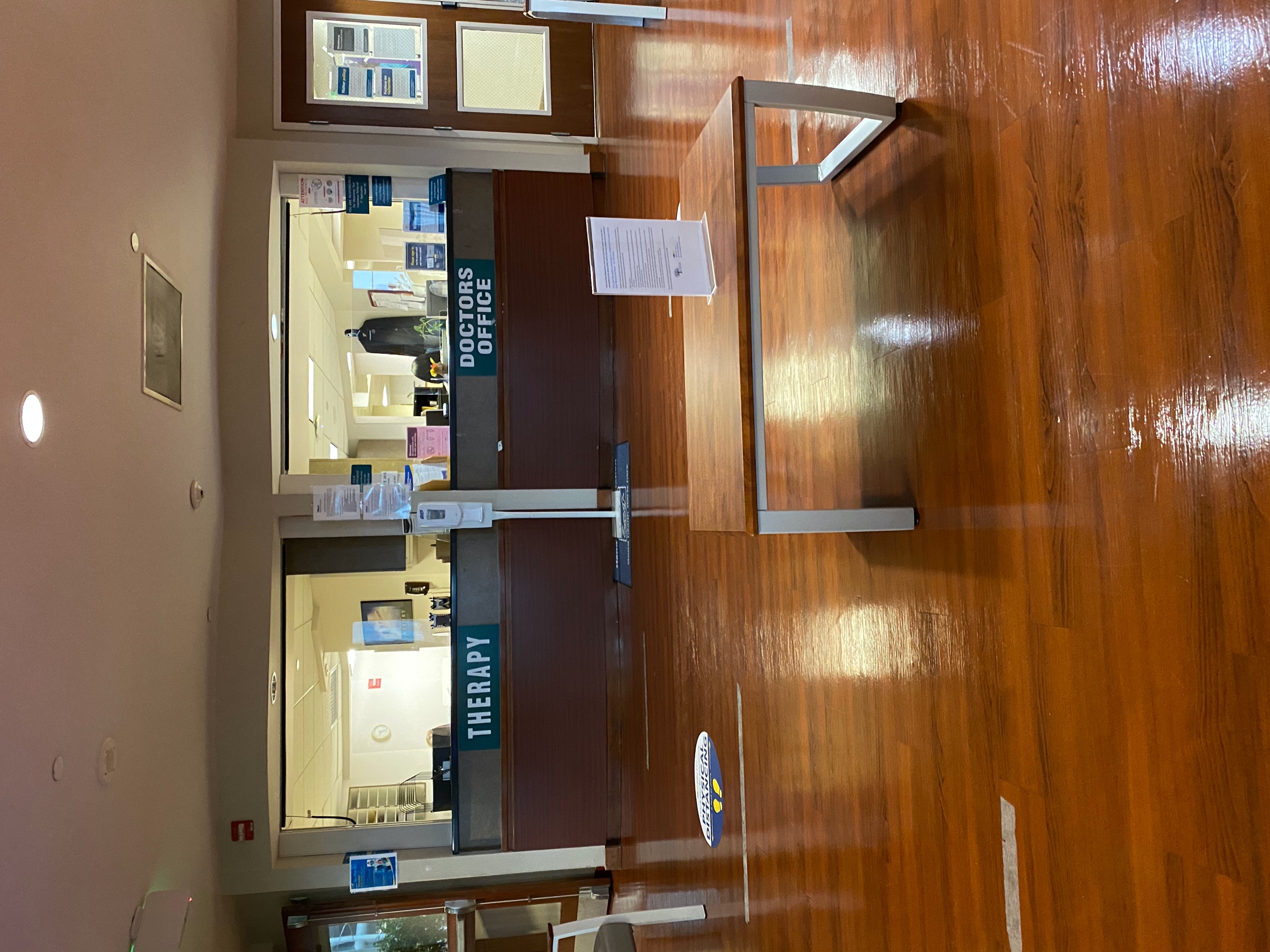


Complete account of the observation:

Summary/write-up including interpretations, reflections, and insights:

- Because of difficulties holding devices, consider mPROM that automatically “plays” audio/video so that they don’t have to click another button; overall might be worthwhile to minimize the number of buttons that they have to press
- Consider mounting iPad on a stand that is wheeled around so that they don’t have to hold the device
- Would need to function both in clinic setting and at home (since patients complete the questionnaires in both settings)
- Benefit of multimedia PROM is that even in a home setting, patients who are low literacy will be able to navigate it (as the patients who do it at clinic benefit from being able to get help from front desk ladies)

## Multimedia PROM Project Direct Observation Data Collection Form - 4

Clinic: Surgeon and therapy clinic / fellow clinic

Location in clinic: CNHC clinic waiting area / check-in counter

Date: 10/27/20

Day of week: Tuesday

The main goals of this data collection are to:

1. Understand the process/flow, routine, and environment of clinic, specifically as it relates to completion of intake surveys and PROMs via the OBERD system

2. Observe scenarios in which patients can or cannot complete intake surveys and PROMs

3. Observe scenarios in which clinic staff need to assist or encourage patients to complete intake surveys and PROMs

4. Use data to inform the PAB meeting and to update the discovery interview guide

5. Use data to generate graphics for use in PAB and future interviews

| **Prompt/ Category** | **What was said?** | **What did you see? What happened? Perception of emotions?** |
| --- | --- | --- |
| Steps of OBERD completion from patient perspective |  |  |
| Patient discussion of OBERD |  |  |
| Staff discussion of OBERD |  |  |
| Patient/ staff interactions surrounding OBERD | 1:10: P: “is your pain mild moderate or severe? Are you satisfied with the movement of your fingers or wrists?” … she continues in this manner, moving down the list of questions, asking them aloud  1:35: Pt: “oh man I filled out so many forms”  1:55: Pt: “I did get your thing in the email.”  D: “yes perfect, I saw that and have printed it out here”  2:09: “What is the level of pain that you have?” “Describe the pain to me” “On a scale of 1-100%, how much % do you think you’re getting out of your hand?” “Is 80% good or bad for you? Is it difficult doing things in your house? Is that always or sometimes that that’s difficult for you? Is your pain mild moderate or severe today? Are you satisfied with the motion in your hand?  2:30: P: “She’s trying to get all your information in the system. I’ll give you these forms and you need to fill these out.”  2:39: Pt: “I filled out a bunch of stuff online…”  2:43: “If I fill out here that my pain feels like a toothache, do you think he’ll get it? It’s just that it hurts all the time” | 1:10: P is helping patient fill out the form. She is reading the questions and some of the responses. He is providing the answers and she is writing it down on a piece of paper. This is needed because he didn’t fill out the forms before arriving for his appointment. Paula said that she chose to assist him with filling out the form (rather than just ask him to use the iPad or ask him to fill out the paper form himself) because she saw that Dr. Baugher has a lot of empty rooms, so she wanted to move things along. Her asking the questions is the most expedient way for the form to get filled out. Sometimes when they’re given the iPad, they sit back down in the waiting room but then start playing on their phones or something, and it takes a while for the OBERD to get filled out.  1:47: P again asking questions, patient is responding, and she is noting them down.  1:55: Think he is referring to his OBERD forms. D has a file and on the top is what appears to be a printed out (and completed) survey.  1:58: another patient is here, to see another doctor—D says he isn’t in the OBERD system at all, because he doesn’t always see patients at this clinic. For this patient, she hands him a paper form to complete. He is a young male (late 20s). Not having any difficulty filling this form out.  There was an issue with his referral; he didn’t have a referral for the appointment from his primary care physician. P gives him a sticky note with an address on it, and tells his PCP to fax the referral. Pt walks out, and in a few minutes walks back in. This is when Paula starts to help him out this form, asking him the questions on the left.  2:25: Another patient is here, and P gives him a form front and back to fill out.  Not sure what P was referring to here  Pt said this as D was taking out a stack of forms |
| Easy patient interactions with OBERD device |  |  |
| Difficult patient interactions with OBERD device |  |  |
| Easy staff interactions with OBERD device |  |  |
| Difficult staff interactions with OBERD device |  |  |
| Other |  |  |

Debriefing notes:

Schematic of clinic space:

Summary/write-up including interpretations, reflections, and insights:

- Many of the decisions surrounding how the OBERD gets completed is driven by what works best for clinic flow and efficiency. How they decide whether to give out the iPad, or give out a paper form, or administer it for the patient is dictated by a number of factors, including: which attending the patient is seeing, whether the patient is on time for the appointment, whether there are open/available patient rooms in the back (indicating that the physician is ready to see more patients), whether there are other patients that need to be helped. It does NOT seem to be decided by whether the patient actually needs help or not.

## Multimedia PROM Project Direct Observation Data Collection Form - 5

Clinic: Surgeon and therapy clinic / fellow clinic

Location in clinic: CNHC clinic waiting area / check-in counter

Date: 10/29/20

Day of week: Thursday

The main goals of this data collection are to:

1. Understand the process/flow, routine, and environment of clinic, specifically as it relates to completion of intake surveys and PROMs via the OBERD system

2. Observe scenarios in which patients can or cannot complete intake surveys and PROMs

3. Observe scenarios in which clinic staff need to assist or encourage patients to complete intake surveys and PROMs

4. Use data to inform the PAB meeting and to update the discovery interview guide

5. Use data to generate graphics for use in PAB and future interviews

| **Prompt/ Category** | **What was said?** | **What did you see? What happened? Perception of emotions?** |
| --- | --- | --- |
| Steps of OBERD completion from patient perspective |  |  |
| Patient discussion of OBERD |  |  |
| Staff discussion of OBERD |  |  |
| Patient/ staff interactions surrounding OBERD |  | 8:54: couple standing at the front, the lady has a splint on her left hand, the guy appears to be the caregiver. D hands him a clipboard; he is the one the holds it and takes it to the waiting room for the both of them. He is the one that takes wallet out and pays with credit card. They walk to back of waiting room where I cannot see them. |
| Easy patient interactions with OBERD device |  |  |
| Difficult patient interactions with OBERD device | 8:57: Clinic staff: “Oh don’t worry about that, you can continue filling it out in the back” | 8:05: pt juggling holding clipboard and pen and phone at the same time, looks like she is struggling a bit, appears that she is looking things up on her phone; struggling as she needs to take the paper out of the clipboard and flip it over to complete the back  8:10: pt stands up and hands the clipboard back to the front desk  8:05: another patient with splint on left hand, is with another person who appears to be a caregiver (wife?), the caregiver is the one with the clipboard who is writing things down and filling out the form. The patient is the one that eventually stands up and hands the clipboard/form back to the front desk. When they are called for appointment, both of them go in together  8:52: they come out from their appointment, are now sitting in waiting area again, waiting for what I believe is their therapy appointment  8:53: called in to therapy side and again both go into the appointment  8:28: patient standing in line, having difficulty putting on his jacket because of a right hand that isn’t working well, now his jacket is only half on and he is only wearing it half on. Holding his right hand/write in left arm. Pt is a therapy patient and goes into therapy.  8:39: another patient here, he stands in line, eventually checks in and walks back to waiting area with a clipboard and pen, his right hand is injured (it is wrapped in ace bandage), but it seems that he is still able to use it with ease. He is filing in the form with his right (injured) hand and holding the clipboard with his left hand. Does not look to be having trouble. Turns over the paper on clipboard and continues filling it out.  8:50: hands form back in  8:52: another couple, man seems to be the one that has the injury, he is also the one that is filling out the clipboard. Woman (caregiver) sitting next to him, not paying attending. He is the one filling out the form  8:57: they are called in for appointment, but he hasn’t finished filling out the form yet. He gets up and walks toward the clinic area, while still filling out the form. |
| Easy staff interactions with OBERD device |  |  |
| Difficult staff interactions with OBERD device |  |  |
| Other |  | There is only one person at front desk today (only D, P is not there) |

Debriefing notes:

Schematic of clinic space:


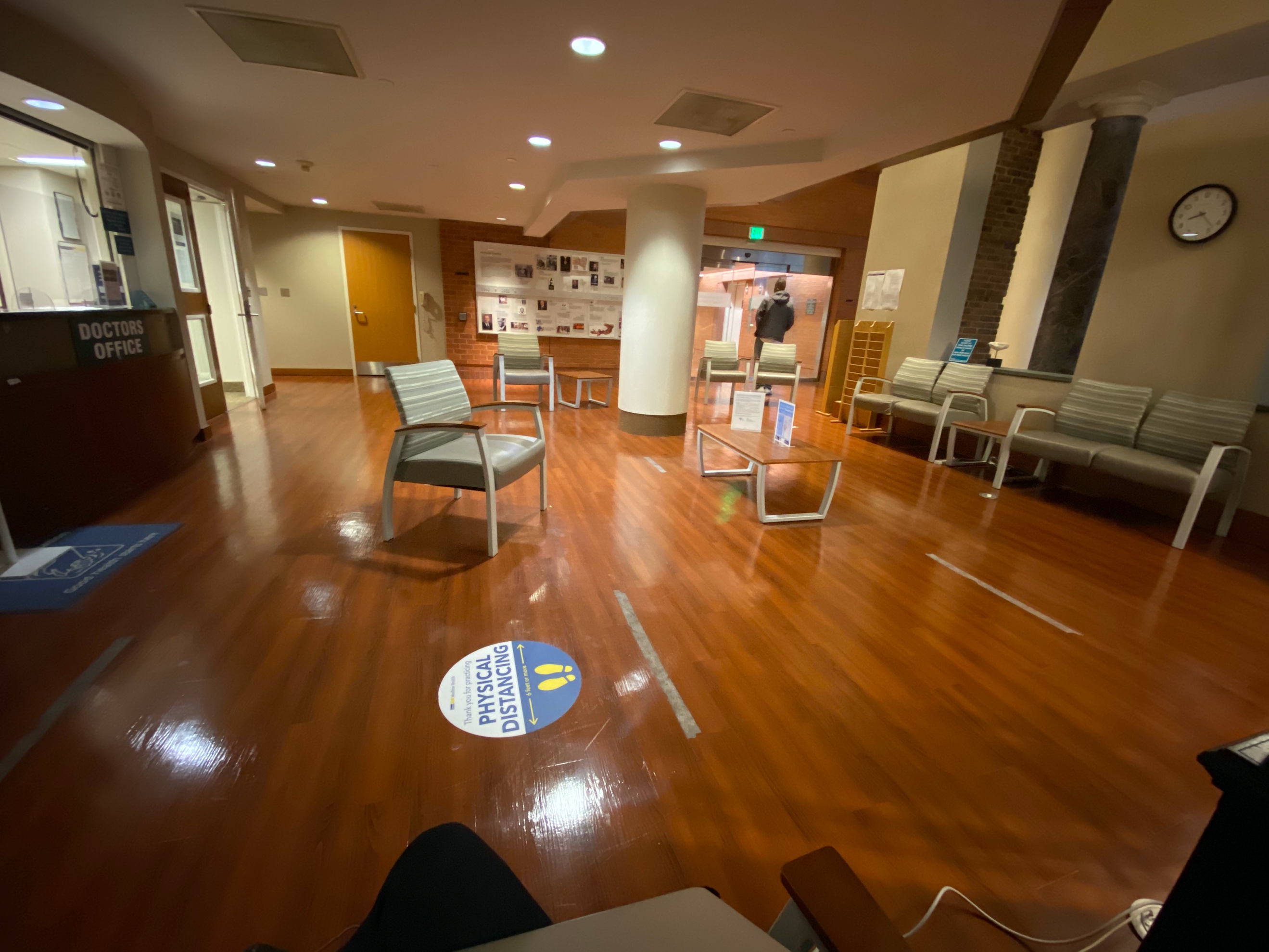


Summary/write-up including interpretations, reflections, and insights:

- Patients sometimes have caregivers with them, demonstrating that caregivers can play an important role in helping the patient fill out forms/surveys.
- Patients have difficulty filling surveys out (in terms of physically maneuvering the survey), whether they’re on paper/clipboard vs. iPad.

## Multimedia PROM Project Direct Observation Data Collection Form - 6

Clinic: Surgeon and therapy clinic / fellow clinic

Location in clinic: CNHC clinic waiting area / check-in counter

Date: 10/29/20

Day of week: Thursday

Whose clinic: Deal

The main goals of this data collection are to:

1. Understand the process/flow, routine, and environment of clinic, specifically as it relates to completion of intake surveys and PROMs via the OBERD system

2. Observe scenarios in which patients can or cannot complete intake surveys and PROMs

3. Observe scenarios in which clinic staff need to assist or encourage patients to complete intake surveys and PROMs

4. Use data to inform the PAB meeting and to update the discovery interview guide

5. Use data to generate graphics for use in PAB and future interviews

| **Prompt/ Category** | **What was said?** | **What did you see? What happened? Perception of emotions?** |
| --- | --- | --- |
| Steps of OBERD completion from patient perspective |  |  |
| Patient discussion of OBERD |  |  |
| Staff discussion of OBERD |  |  |
| Patient/ staff interactions surrounding OBERD |  |  |
| Easy patient interactions with OBERD device |  | 1:04: pt filling out form on clipboard, he is young, there is someone who seems to be a parent or a caregiver who is helping him fill the form out, he asked Siri on his phone something, his phone responded “penicillin is a class of drugs.” Finishes filling out form and turns it in. The two people then sit separately, it isn’t clear why they aren’t sitting next to each other.  1:14pm: who looked to be the caregiver is now filling out how own form. Perhaps these 2 people aren’t together. As he is filling out form, he is continually talking into his phone. Can’t hear what he is saying. Not sure if someone is on the other end. Doesn’t appear that he is having trouble filling the form out.  1:18pm: gives the form back to the front desk |
| Difficult patient interactions with OBERD device |  |  |
| Easy staff interactions with OBERD device |  |  |
| Difficult staff interactions with OBERD device |  |  |
| Other |  | There are again 2 ladies checking people in, at the check in area |

Debriefing notes:

- Things really slowed down! From 1:45-2:00pm, there weren’t any new patients coming; seems that overall patient volume is lower on this side
- No iPads were given out

Schematic of clinic space:


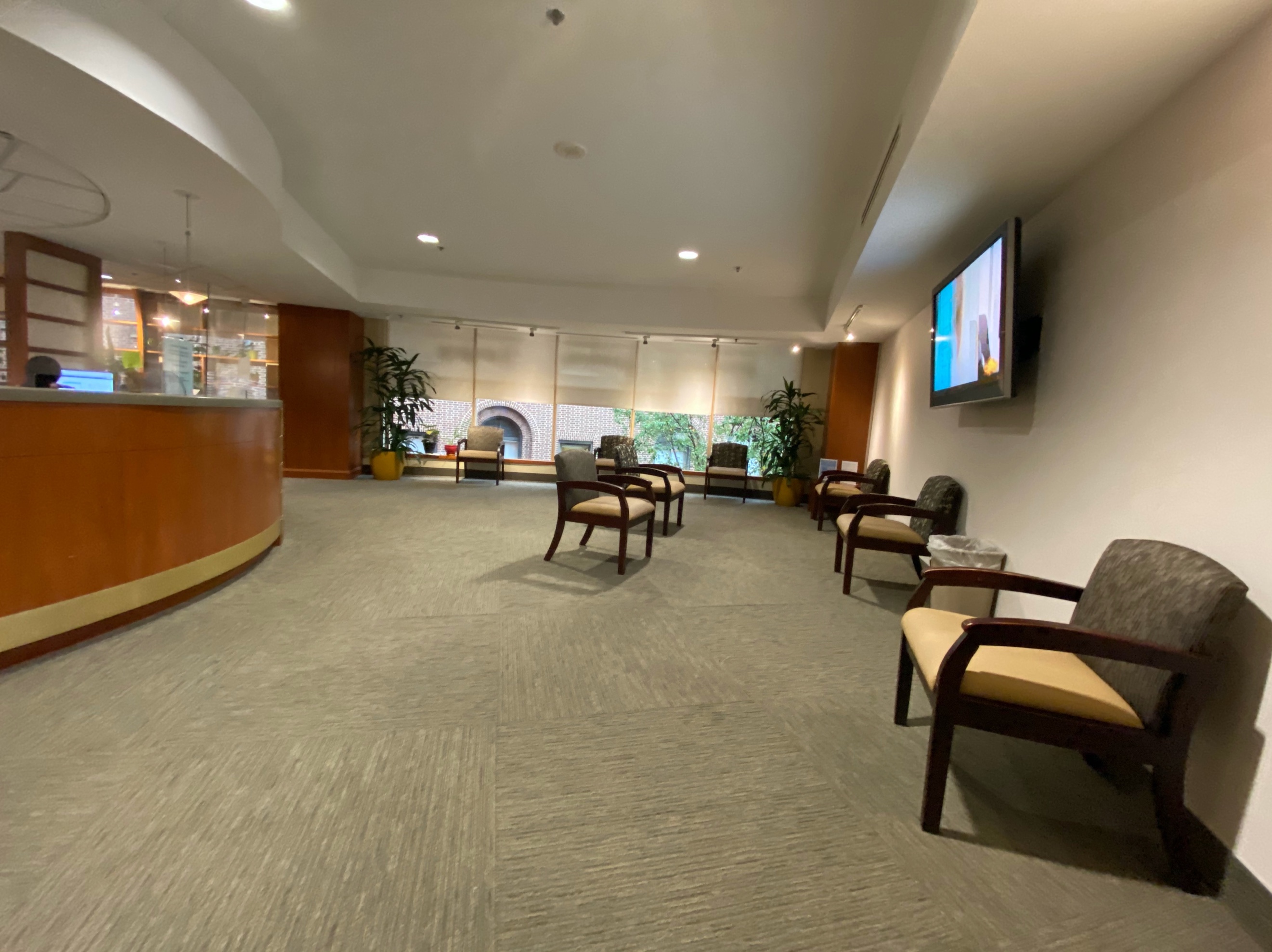


Summary/write-up including interpretations, reflections, and insights:

- Many of the patients have a caregiver with them. Will be important to incorporate them as an additional stakeholder group.
- Most of the patients fill out forms on a clipboard; there are no iPads given out/used during the observation.

# DISCOVERY INTERVIEW GUIDES

## Target Participant: Patients

Background: There will be three parts to this interview, utilizing either the semi-structured interview or the cognitive interview. The goals of the semi-structured interviews are to: Understand perceptions of and challenges surrounding 1) how PROMs are completed (i.e., process) and 2) the platform on which they are administered (i.e., delivery). The goals of the cognitive interview are to: Understand how patients perceive and interpret each PROMIS UE question and response (i.e., content). The interview ends with a literacy screen.

Introduction

- Thank you so much for agreeing to participate in this study.
- Would it be OK for me to audio record our conversation? This recording will only be used to remind me what we have talked about today. It will not be shared with anyone outside of our research group.
- There are no right or wrong answers to the questions I’m going to ask you. I would just ask you to speak as you would naturally and tell me as much as you can about your experiences. I am really looking forward to learning from you today.

Semi-structured interview on PROM process: These (*show PROMs on iPad*) are some of the surveys that we ask patients to complete. You may have been asked to complete them at home before your appointment or you may have been asked to complete them at the clinic.

1. Can you please tell me about any experiences that you have had with these surveys?
2. How did you feel about the process of completing the survey?
   - Probe: Bored? Interested? Annoyed? Skeptical? Confused? That your perspectives and opinions about your health were being heard?
   - What made you feel that way?
3. Some patients don’t complete the survey every time. Did you complete the survey at your most recent appointment?
   - Follow-up if no: Some patients don’t complete the survey. What led you to not complete it?
4. Where did you complete the survey?
   - Why did you complete it there?
   - Why did you not complete it [at home/at clinic]?
5. Did you complete it alone or with someone else?
   - If someone else, who? Why did you complete it with that person?
   - What did that person do?
     - - Did that person record your responses for you? If yes, did you feel that that person recorded your responses accurately?
   - Would you ask that person for help again?
   - If alone, was there anyone that you would’ve liked to complete it with?
     - - If yes: who? Why that person?
       - If no: why do you prefer to complete it alone?
6. Were there parts or aspects of completing the survey that you could have used help with?
   - If yes: which parts or aspects?
   - If yes: who would you have liked the help from? Who could have helped you?
7. You were asked to complete several surveys. What did you think about the number of surveys that you were asked to complete?
   - Did it take too long to complete? Or did you think it was reasonable?
   - Do you think you gave equal attention to the earlier surveys vs. the later ones?
8. How do you think these survey results are used?
   - Do you think that completing these forms are important, or do they feel more like a nuisance?
   - If important: what about these forms made you think that they’re important to complete?
   - If nuisance: why don’t you feel that it is important to complete these forms?

Semi-structured interview on PROM delivery: Now I’d like to ask some questions about the method that you use to complete the survey. This will help us improve the way that the survey is administered.

1. [If completed at home]: what device did you complete it on? [If completed at clinic]: did you complete the form on an iPad, on paper, or did a clinic staff help you complete it on their computer?
2. How did that go?
3. Why did you choose that method?
4. Was there any confusion or uncertainty about how to complete it?
5. Did you have any physical difficulties with completing the form?
   - For example, does your condition make it harder to use an iPad or a pen/paper?
6. In the future, would you prefer to complete the surveys yourself, or to have the clinic staff administer it? Why?
7. A few of the possible platforms we use to administer surveys include paper, iPad, smartphone, laptop, and computer. Which of these are you familiar with? Which method do you find the easiest for you?

- Why that method?
- What do you (think you would) find challenging about the other methods?
- Do you prefer paper or digital methods?
- Of the digital methods, which do you prefer the most, and why?

Cognitive interview on PROMIS UE content: I’d like to transition to focusing on the questions from one specific survey. (*Give patient paper PROMIS UE.*) We know that no survey is perfect. We’d like to learn more about how people understand and respond to the questions in this survey, so we can improve it. A way we find out more about the questions is to hear what people are thinking about the questions as they answer them. So, I’d like you to read the question silently to yourself. Then, I’d like you to say aloud what you’re thinking as you’re reading the question and what goes through your mind as you decide how to answer. We are interested in what you say to yourself as you read and respond to the questions. In order to do this, we will ask you to THINK ALOUD. I want you to say out loud everything that you say to yourself silently. Just speak as if you are alone in the room speaking to yourself. I will then ask you some follow-up questions. So, let’s start with the first question. (*Point to the first question on the paper. Repeat this and steps 18-20 for each PROMIS UE question*.)

1. Please can you tell me, in your own words, what is this question asking?
   - Do you think there is anything else it may mean?
   - Are there any words or parts of the question that are unclear?
   - What we meant by this question is ….. Do you have any suggestions for how we could re-write the question so that it is clearer?
2. Did you have to “guess” or choose a random answer on this question? If yes, why?
   - Did you feel that you needed any help answering this question?
3. How did you feel about answering this question?
   - Did you find this question challenging or easy? What made it challenging or easy?
4. [Only ask after 1^st^ PROMIS question]: Before we move to the second question, I’m curious about your thoughts about these responses, rather than the questions. What did you think about the response options available on this survey?

- In your own words, can you tell me what each response means?
- Did you feel that you had enough, too many, or just the right number of options?

Follow-up questions from cognitive interview (to be asked after all PROMIS UE questions have been covered):

1. Some surveys use images or illustrations. Are there any questions on this survey that you think would be more clear with an image or an illustration?
   - If yes, which question(s)?
   - If yes, what would you include an image or illustration of?
   - If no, why not?
2. What about including a video? Are there any questions on this survey that you think would be more clear with a video?
   - If yes, which question(s)?
   - If yes, what would you include a video of?
   - If no, why not?
3. What about including an audio clip?
   - If yes, which question(s)?
   - If no, why not? What would you choose to include instead, if anything?

Literacy screen:

1. In the final part of the interview, I would like to see which of these words you are familiar with. (*Show list of REALM-R words, and point to the first word*). Please say all of the words you know. If you come to a word you do not know, you can sound it out or just skip it and go on.

Closing remarks

- This brings our interview to a close. This was incredibly helpful. Is there anything else you’d like to share that I didn’t ask?
- Thank you for your time and for your thoughts. I really appreciate all the insight that you provided.

## Target Participant: Caregivers

Background: There will be two parts to this interview, both utilizing the semi-structured interview. The goals are to: Understand perceptions of and challenges surrounding 1) how PROMs are completed (i.e., process) and 2) the platform on which they are administered (i.e., delivery).

Introduction

- Thank you so much for agreeing to participate in this study.
- Would it be OK for me to audio record our conversation? This recording will only be used to remind me what we have talked about today. It will not be shared with anyone outside of our research group.
- There are no right or wrong answers to the questions I’m going to ask you. I would just ask you to speak as you would naturally and tell me as much as you can about your experiences. I am really looking forward to learning from you today.

Questions about the PROs process:

1. First, what is your relationship to *** (patient’s name)?
2. Because you accompanied *** to his/her appointment, you may have seen that we ask our patients to complete surveys before their appointment. Can you walk me through how *** completes these surveys?
   - Probe: The surveys can be completed either at home or in clinic, before the appointment. Does *** complete them at home or in clinic?
   - Why do you think *** chooses to complete them there?
   - What do you see as the advantages or disadvantages in each of those settings?
3. What is your impression of this process?
   - Probe: How do you feel about this process?
   - Probe: Easy? Challenging? Frustrating? Seamless?
4. What role, if any, did you play in ***’s completion of these surveys?
   - Probe: Did you need to help ***?
     - - If yes, in what capacity?
       - If not, did you think *** could have benefited from your help?
5. What are some challenges, if any, that YOU have encountered while playing that role?
   - [If there are challenges]: How did you overcome those challenges?
6. How do you think *** (patient’s name) feels about the surveys?
   - What makes you think that they feel this way?
7. What are some challenges, if any, that you have witnessed *** having with the process?
   - [If there are challenges]: How were these challenges overcome?
8. Can you remember any instances in which *** had trouble reading or understanding what the survey was asking?
   - If yes: Can you tell me more about those instances?
9. What changes can you think of, if any, that would have made it easier for *** to complete these surveys?
10. Have you ever completed forms like these for your own medical appointments?
    - If yes: Please tell me about that experience
    - If yes: How was it different?
      - - Probe: What was better or worse?
11. What would be a better way to get this information?
12. Can you think of anything that would make survey completion more helpful, impactful, or beneficial for you or ***?
13. Do you think that asking patients to complete these surveys is important, or more of a nuisance?
    - If important: what about these surveys make you think that they’re important to complete?
    - If nuisance: why don’t you feel that it is important for patients to complete these surveys?

Questions about PROs delivery: Now I’d like to ask some questions about the method that *** used to complete the survey.

1. Did *** complete the surveys on paper, on an iPad tablet, on the computer, on a phone, or a combination of those?
2. For that (those) platform(s), how did it go?
   - Probe: With that platform, was there any confusion or uncertainty about how to complete it?
   - If yes: can you tell me more about those instances?
   - If yes: how was this overcome?
   - If yes: did you play a role in overcoming this?
3. For that (those) platform(s), did *** have physical difficulties with completing it?
   - If yes: can you tell me more about those instances?
   - If yes: how was this overcome?
   - If yes: did you play a role in overcoming this?
4. Of the platforms that we’ve discussed, which do you think would be best for ***?
   - Why that one?
5. What is your comfort level with technology?
   - If the survey were on a computer, or a tablet, or a phone, would you be able to help the patient navigate it?

Concluding questions:

1. Some surveys use images or illustrations. Do you think including these would be helpful for ***?
   - Why or why not?
2. What about including a video?
   - Why or why not?
3. What about including an audio clip?
   - Why or why not?
4. If you could improve either the surveys themselves or the process by which patients take them, how would you improve it?
   - Probe: What, if anything, would you change about the way that we currently deliver surveys?
   - Probe: What, if anything, would you change about the survey itself?

Closing remarks

- That is all I had on my list to review with you today. This was incredibly helpful. Is there anything else you’d like to share that I didn’t ask?
- Thank you for your time and for your thoughts. I really appreciate all the insight that you provided.

## Target Participant: Clinic Staff

Background: There will be two parts to this interview, both utilizing the semi-structured interview. The goals are to: Understand perceptions of and challenges surrounding 1) how PROMs are completed (i.e., process) and 2) the platform on which they are administered (i.e., delivery).

Introduction

- Thank you so much for agreeing to participate in this study.
- Would it be OK for me to audio record our conversation? This recording will only be used to remind me what we have talked about today. It will not be shared with anyone outside of our research group.
- There are no right or wrong answers to the questions I’m going to ask you. I would just ask you to speak as you would naturally and tell me as much as you can about your experiences. I am really looking forward to learning from you today.

Semi-structured interview on PROM process: As you know, we ask all of our patients to complete surveys before their appointment.

1. First, can you walk me through how patients complete these surveys?
   - Probe: Can you walk me through the different scenarios in which patients take their surveys?
2. What is your impression of this process?
   - Probe: How do you feel about this process?
   - Probe: Easy? Challenging? Frustrating? Worthwhile?
3. What are some challenges, if any, that YOU have with the process?
   - [If there are challenges]: How do you overcome those challenges?
4. How do you think patients feel about the surveys?
   - What makes you think that they feel this way?
5. What are some challenges, if any, that PATIENTS have with completing the surveys?
   - Probe: In this study, we are specifically interested in how we can improve this process for low literacy patients. Can you remember any instances in which patients had trouble reading or understanding what the survey was asking?
   - [If there are challenges]: How do they overcome those challenges?
6. I’ve noticed that there are times when you administer the survey to the patient. How do you feel about this process?
   - Can you help me understand when you choose to do this?
7. I’ve noticed that there is oftentimes someone who accompanies the patient to their appointment. In what ways, if any, have you seen the caregiver interact with the survey completion process?
8. How do you think these survey results are used?
   - Do you think that asking patients to complete these surveys is important, or more of a nuisance?
   - If important: what about these surveys make you think that they’re important to complete?
   - If nuisance: why don’t you feel that it is important for patients to complete these surveys?

Semi-structured interview on PROM delivery: Now I’d like to ask some questions about the method that patients use to complete the survey.

1. I’ve noticed that there are times when the patient completes the survey either on the iPad or on paper. Can you help me understand what determines which one they get?
2. How does it usually go when you give them the iPad?
3. How does it usually go when you give them the paper?
4. With any of the methods, is there confusion or uncertainty about how to complete it?
   - If yes: what caused it?
5. With any of the methods, do patients have physical difficulties with completing it?
   - If yes: what caused it?
   - For example, does their condition make it harder to use an iPad or a pen/paper?
6. If you had to redesign the way that we deliver these surveys, how would you design it?
   - What would you change about the way that we currently deliver surveys?
7. Some surveys use images or illustrations. Do you think including these would be helpful?
   - Why or why not?
8. What about including a video?
   - Why or why not?
9. What about including an audio clip?
   - Why or why not?
10. Of the methods that we use in clinic now (paper, iPad, or your computer), which do you prefer the most?

- Why that method?

Closing remarks

- This brings our interview to a close. This was incredibly helpful. Is there anything else you’d like to share that I didn’t ask?
- Thank you for your time and for your thoughts. I really appreciate all the insight that you provided.

# IDEATION WORKSHOP OUTLINE

**Participants:** Chao Long, Claudia Udler, Aviram Giladi, Laura Beres, Albert Wu, Hayelin Choi

**Goals:**

- Review research insights, challenges, and opportunities
- Engage the research team in brainstorming session
- Identify initial ideas
- Develop an abstraction of what first prototype(s) will include

**Preparation before workshop:**

- To be done by Chao
  - Compile insights document
  - Compile opportunities outline document
  - Send to participants before the workshop: Zoom invite, insights document, opportunities outline document, brainstorming worksheet (and ask them to print out 10 copies of this)
  - Make slides for Part I
- To be done by Claudia
  - Make slides for Part II
- To be done by both Claudia and Chao
  - Review and edit all slides
- To be done by everyone:
  - Print out brainstorming worksheet
  - Read the insights document and the opportunities outline

**Roles during workshop**:

- Claudia: Facilitator
  - Timekeeper
  - The stage manager (slides)
- Chao: Host
  - Man Miro, the interactive “whiteboard”
- All others
  - Part I: listen to the findings and context
  - Part II: complete the Brainstorming Worksheet and participate in the group activities

**Agenda:**

- RECORD MEETING
- **PART I: Introduction & Presentation (led by Chao) – 45 min**
  - **5 min:** Welcome
    - Brief introductions by all
    - Tell everyone the meeting will be recorded; get their consent
    - Briefly introduce agenda for workshop
  - **10 min:** Intro / context for project
    - Outline goals for the meeting (why we’re here, what we have done so far, the goal of the day). We would like to walk away with ideas (I like, I wish, What if)
    - Be clear what we want to walk away with and what the components of that include (i.e., not the specific image, what how the image is delivered, etc)
    - Brief reminder of PROMIS instrument and the 7 questions
  - **30 min:** Research share back: a comprehensive summary of insights, interview summary, personas, and opportunities outline discussion. (Opportunities discussion: where do we go from here? Themes > Insights> Opportunities/Challenges)
    - Include boundaries
- **PART II: Group work (led by Claudia) – 1h15 min**
  - **10 min:** Go over/review the steps in the Brainstorming Worksheet, provide an example, and do a warm-up exercise
  - **10 min:** Brainstorm ideas individually; each participant is assigned a different theme; each participant comes up with many ideas for their theme
  - **15 min:** Each individual shares their ideas for their assigned theme. For each theme, the entire group adds/brainstorms additional ideas
  - **10 min**: Identify the best/top idea(s) per theme; vote on these ideas as a group to decide what should be used for the prototype (these ideas should be very detailed and can be operationalized)
  - **15 min**: Assemble the ideas voted on by the group into a prototype
  - **15 min:** Determine which components of the prototype need to be tested and what is the best way to test them
- **WRAP-UP (Claudia, 5 min):** Wrap-up, brief explanation of next steps of process

## Ideation Worksheet


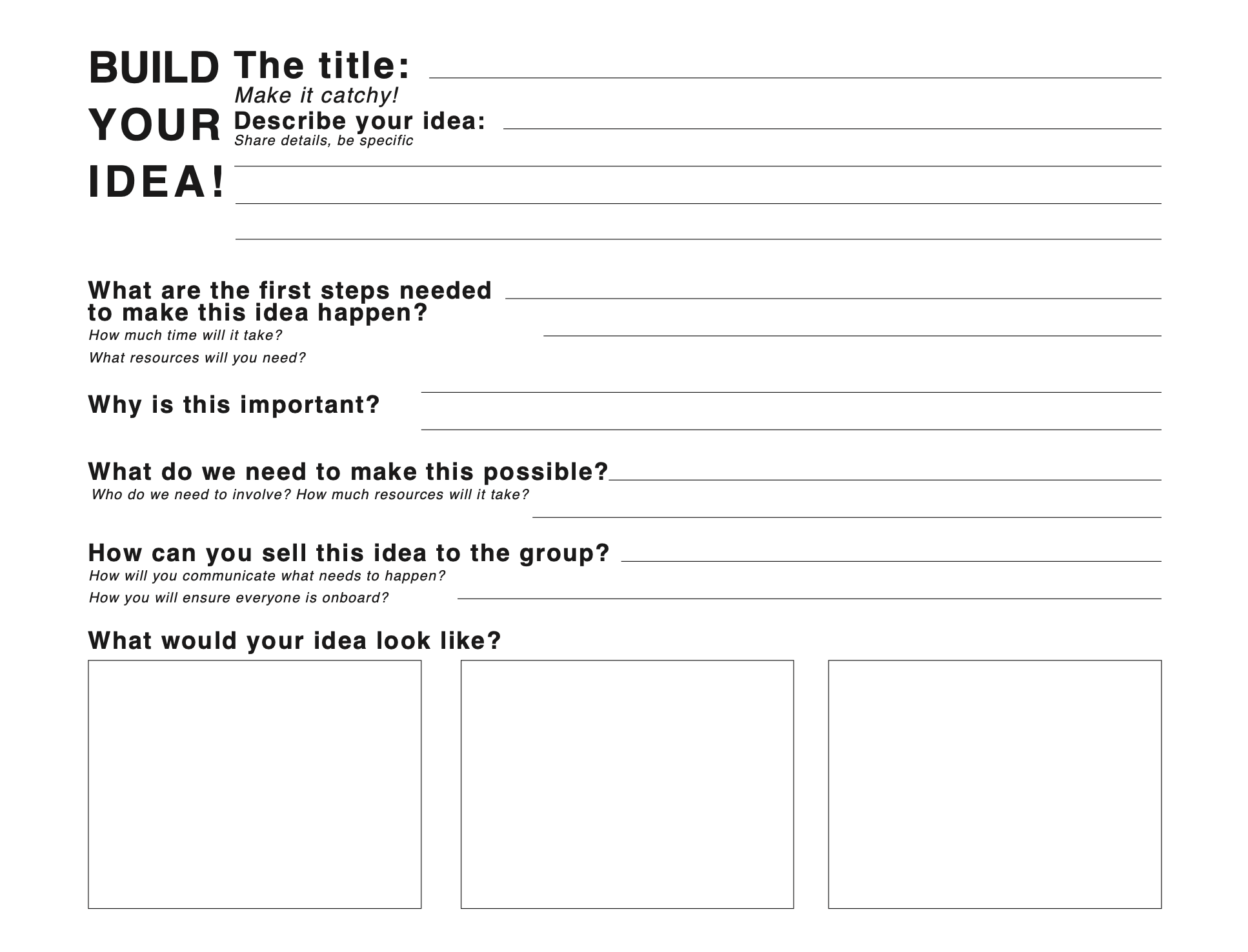


## Ideation Worksheet Example


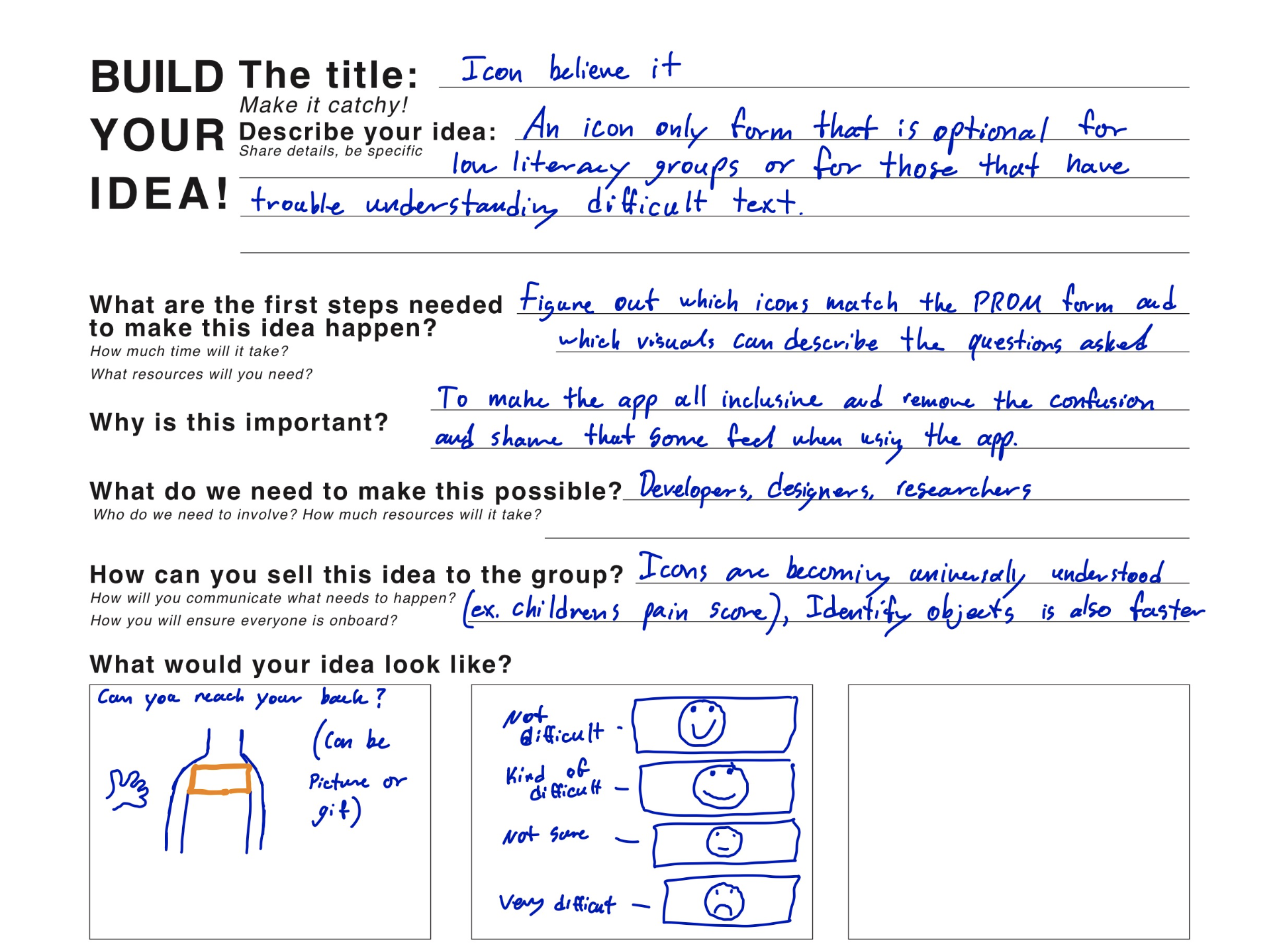


# MEMBER CHECKING INTERVIEW GUIDE

Parts to this interview:

1. Discovery interview
2. Cognitive interview
3. Member checking
4. Literacy screen

Introduction

- Thank you so much for agreeing to participate in this study.
- Would it be OK for me to audio record our conversation? This recording will only be used to remind me what we have talked about today. It will not be shared with anyone outside of our research group.
- There are no right or wrong answers to the questions I’m going to ask you. I would just ask you to speak as you would naturally and tell me as much as you can about your experiences. I am really looking forward to learning from you today.

Discovery interview on PROM completion: You may have been asked to complete them at home before your appointment or you may have been asked to complete them at the clinic.

1. Can you please tell me about any experiences that you have had with these surveys?
2. How did you feel about the process of completing the survey?
   - Probe: Bored? Interested? Annoyed? Skeptical? Confused? That your perspectives and opinions about your health were being heard?
   - What made you feel that way?
3. Some patients don’t complete the survey every time. Did you complete the survey at your most recent appointment?
   - Follow-up if no: Some patients don’t complete the survey. What led you to not complete it?
4. Where did you complete the survey?
   - Why did you complete it there?
   - Why did you not complete it [at home/at clinic]?
5. Did you complete it alone or with someone else?
   - If someone else, who? Why did you complete it with that person?
   - What did that person do?
     - - Did that person record your responses for you? If yes, did you feel that that person recorded your responses accurately?
   - Would you ask that person for help again?
   - If alone, was there anyone that you would’ve liked to complete it with?
     - - If yes: who? Why that person?
       - If no: why do you prefer to complete it alone?
6. Were there parts or aspects of completing the survey that you could have used help with?
   - If yes: which parts or aspects?
   - If yes: who would you have liked the help from? Who could have helped you?
7. You were asked to complete several surveys. What did you think about the number of surveys that you were asked to complete?
   - Did it take too long to complete? Or did you think it was reasonable?
   - Do you think you gave equal attention to the earlier surveys vs. the later ones?
8. How do you think these survey results are used?
   - Do you think that completing these forms are important, or do they feel more like a nuisance?
   - If important: what about these forms made you think that they’re important to complete?
   - If nuisance: why don’t you feel that it is important to complete these forms?

Discovery interview on PROM delivery: Now I’d like to ask some questions about the method that you use to complete the survey. This will help us improve the way that the survey is administered.

1. [If completed at home]: what device did you complete it on? [If completed at clinic]: did you complete the form on an iPad, on paper, or did a clinic staff help you complete it on their computer?
2. How did that go?
3. Why did you choose that method?
4. Was there any confusion or uncertainty about how to complete it?
5. Did you have any physical difficulties with completing the form?
   - For example, does your condition make it harder to use an iPad or a pen/paper?
6. In the future, would you prefer to complete the surveys yourself, or to have the clinic staff administer it? Why?
7. A few of the possible platforms we use to administer surveys include paper, iPad, smartphone, laptop, and computer. Which of these are you familiar with? Which method do you find the easiest for you?

- Why that method?
- What do you (think you would) find challenging about the other methods?
- Do you prefer paper or digital methods?
- Of the digital methods, which do you prefer the most, and why?

Cognitive interview on PROMIS UE content: I’d like to transition to focusing on the questions from one specific survey. (*Give patient paper PROMIS UE.*) We know that no survey is perfect. We’d like to learn more about how people understand and respond to the questions in this survey, so we can improve it. A way we find out more about the questions is to hear what people are thinking about the questions as they answer them. So, I’d like you to read the question silently to yourself. Then, I’d like you to say aloud what you’re thinking as you’re reading the question and what goes through your mind as you decide how to answer. We are interested in what you say to yourself as you read and respond to the questions. In order to do this, we will ask you to THINK ALOUD. I want you to say out loud everything that you say to yourself silently. Just speak as if you are alone in the room speaking to yourself. I will then ask you some follow-up questions. So, let’s start with the first question. (*Point to the first question on the paper. Repeat this and steps 18-20 for each PROMIS UE question*.)

1. Please can you tell me, in your own words, what is this question asking?
   - Do you think there is anything else it may mean?
   - Are there any words or parts of the question that are unclear?
   - What we meant by this question is ….. Do you have any suggestions for how we could re-write the question so that it is clearer?
2. Did you have to “guess” or choose a random answer on this question? If yes, why?
   - Did you feel that you needed any help answering this question?
3. How did you feel about answering this question?
   - Did you find this question challenging or easy? What made it challenging or easy?
4. [Only ask after 1^st^ PROMIS question]: Before we move to the second question, I’m curious about your thoughts about these responses, rather than the questions. What did you think about the response options available on this survey?

- In your own words, can you tell me what each response means?
- Did you feel that you had enough, too many, or just the right number of options?

Follow-up questions from cognitive interview (to be asked after all PROMIS UE questions have been covered):

1. Some surveys use images or illustrations. Are there any questions on this survey that you think would be more clear with an image or an illustration?
   - If yes, which question(s)?
   - If yes, what would you include an image or illustration of?
   - If no, why not?
2. What about including a video? Are there any questions on this survey that you think would be more clear with a video?
   - If yes, which question(s)?
   - If yes, what would you include a video of?
   - If no, why not?
3. What about including an audio clip?
   - If yes, which question(s)?
   - If no, why not? What would you choose to include instead, if anything?

Member checking: We’ve interviewed some other patients already, and have started to draw some conclusions from what they’ve told us. In this next part, I’d like to share some of those, to see if those sound about right to you.

1. Customizability: Some patients think images/videos/audio might help, while others don’t think they’d help. So, we think it might be best to design one instrument that can be changed and customized for different patients, so that each patient can pick and use whatever version they prefer or need. Does that sound like a good idea to you?
   - Why or why not?
2. Caregivers: We think that even though there are caregivers who play a role in caring for a patient, some caregivers will be able to help the patient complete the instrument, while others won’t be able to. Does that sound about right to you?
   - Why or why not?
3. Barriers: We identified several barriers to completing the instrument. These included difficulty reading, difficulty with the technology, difficulty with English, physical difficulties due to a hand condition, or difficulty with vision. Does that sound about right to you?
   - Why or why not?
   - Are there other barriers that you can think of that we haven’t identified?
4. Ambiguity: We think that some of the questions on the instrument can be confusing or can be interpreted in different ways. Patients may answer the questions differently depending on exactly how they are interpreting it. Do you agree with that?
   - We think that including a gif that is acting out the question might help patients better understand *exactly* what the question is asking. Do you agree with that?
   - Why or why not?
5. Purpose: We think that it might be helpful to include a brief explanation of the purpose of the survey, and how the results will be used, at the beginning. Do you agree?
   - Why or why not?

Literacy screen:

1. In the final part of the interview, I would like to see which of these words you are familiar with. (*Show list of REALM-R words, and point to the first word*). Please say all of the words you know. If you come to a word you do not know, you can sound it out or just skip it and go on.

Closing remarks

- This brings our interview to a close. This was incredibly helpful. Is there anything else you’d like to share that I didn’t ask?
- OK to contact again for future testing?
- Thank you for your time and for your thoughts. I really appreciate all the insight that you provided.
