# Supplementary material for: Developing a multimedia patient-reported outcomes measure for low literacy patients with a human-centered design approach
Source: PLoS One. 2024 Jun 5;19(6):e0304351. doi: 10.1371/journal.pone.0304351 (PMC11152264; doi:10.1371/journal.pone.0304351)
Supplement: Attachment — Submitted filename: Table of corrections PONE-D-23-24670_Final.doc [file pone.0304351.s002.doc]

**PONE-D-23-24670 - Developing a multimedia patient-reported outcomes measure for low literacy patients**

| **Numbered Reviewer Remark and Manuscript Line Number** | **Author Response** | **Revised Manuscript Line Number and Text Change** |
| --- | --- | --- |
| Journal Requirements | | |
| 1. Please ensure that your manuscript meets PLOS ONE's style requirements, including those for file naming. | Thank you. The file names have been updated. |  |
| 2. In your Data Availability statement, you have not specified where the minimal data set underlying the results described in your manuscript can be found. PLOS defines a study's minimal data set as the underlying data used to reach the conclusions drawn in the manuscript and any additional data required to replicate the reported study findings in their entirety. All PLOS journals require that the minimal data set be made fully available. For more information about our data policy, please see http://journals.plos.org/plosone/s/data-availability [journals.plos.org].  Upon re-submitting your revised manuscript, please upload your study’s minimal underlying data set as either Supporting Information files or to a stable, public repository and include the relevant URLs, DOIs, or accession numbers within your revised cover letter. For a list of acceptable repositories, please see http://journals.plos.org/plosone/s/data-availability#loc-recommended-repositories [journals.plos.org]. Any potentially identifying patient information must be fully anonymized.  Important: If there are ethical or legal restrictions to sharing your data publicly, please explain these restrictions in detail. Please see our guidelines for more information on what we consider unacceptable restrictions to publicly sharing data: http://journals.plos.org/plosone/s/data-availability#loc-unacceptable-data-access-restrictions [journals.plos.org]. Note that it is not acceptable for the authors to be the sole named individuals responsible for ensuring data access.  We will update your Data Availability statement to reflect the information you provide in your cover letter. | Thank you for bringing this to our attention. We have compiled our study’s minimal underlying data set as a Supporting Information file. This file includes direct observation data, interview guides, and workshop outlines.  Of note, the interview audio recordings cannot be publicly shared because these data cannot be fully de-identified and anonymized. Specifically, certain interviews mention details that make it possible for patients to be identified. It is also possible for patients to be identified based on their voice. Some interviews also contain sensitive patient information. These data may be requested from Isaac C. Fleming, who can be reached at [Ike.C.Fleming@Medstar.net](mailto:Ike.C.Fleming@Medstar.net). |  |
| 3. We note that Figure 1 in your submission contain copyrighted images. All PLOS content is published under the Creative Commons Attribution License (CC BY 4.0), which means that the manuscript, images, and Supporting Information files will be freely available online, and any third party is permitted to access, download, copy, distribute, and use these materials in any way, even commercially, with proper attribution. For more information, see our copyright guidelines: http://journals.plos.org/plosone/s/licenses-and-copyright [journals.plos.org]. | The Figure 1 submitted in the original submission does not include any copyrighted images. |  |
| Reviewer 1 | | |
| 1. There is no description of the patient population, other than to state that they had “a hand and upper extremity condition.” It is not clear what this means. Also, how large is this population in the U.S.? | Thank you for bringing this to our attention. We agree that this is not clear as stated and have updated the manuscript to provide more details about the patient population. In the US in 2010, 2.8 million patients were treated in the ED for hand/upper extremity conditions, although this does not capture the outpatient volume (Wenzinger et al. Trends in Upper Extremity Injuries Presenting to US Emergency Departments. Hand (N Y). 2017). | Line 155-159: “We recruited adult patients with any hand and upper extremity condition including but not limited to those that are traumatic, infectious, congenital, oncologic, and rheumatologic in nature because this is the population for which the PROMIS Upper Extremity is validated in. Patients who identified as English-speaking and who had previously completed the PROMIS-UE were eligible for recruitment.”  Lines 237-238: “Table 2 provides the demographics and characteristics for our entire cohort of 17 participants.” |
| 2. What mode and method of administration were used for the direct observation phase of PROM administration and completion in the clinic’s waiting and check-in areas? | Thank you for your question. PROMs were either self-administered or administered by clinic staff. PROMs were completed either with paper-and-pencil or via a tablet, depending on patient preference. We agree that these are important details and have added them to our manuscript. | Lines 144-145: “PROMs were either self-administered or administered by clinic staff, via paper-and-pencil or tablet depending on patient preference.” |
| 3. The author chose the Patient Reported Outcomes Measurement Information System Upper Extremity Short Form 7a (PROMIS-UE). A definition of this measure is needed. | Thank you for pointing this out. This has been added. | Lines 113-114: “The PROMIS-UE is a disease-specific measure validated to capture patient perception of upper extremity physical status or ability.” |
| Reviewer #2 | | |
| This well-written paper essentially describes a human-centered design process. On the whole, it is overly focused on methods (HCD) and not focused enough on results. In the abstract, the first five sentences of "results" belong with the methods. The development of the mPROMIS-UE prototype is the result - not the "identification" of personas. ("Identification" seems an overly strong word choice. My impression is that your observations and interviews informed the profiles and personas that you used to develop the modified tool.) | Thank you for your comment. We believe that the findings from each stage of our HCD process are more appropriate as “results” rather than as “methods.” For example, “direct observation” is the method, while analysis of what was observed yields the “results,” in this case the “personas.” We believe that this framing and categorization of methods versus results is consistent with what is standard for scientific manuscripts that employ a HCD process. This is supported by the following reference: Bazzano AN, Yan SD, Martin J, et al. Improving the reporting of health research involving design: a proposed guideline. BMJ Global Health 2020;5:e002248. Doi:10.1136/bmjgh-2019-002248. Specifically, Bazzano et al. recommends that the “results” include a “summary of findings from design activities,” which we believe the current manuscript reflects. There are also numerous examples of published papers employing a HCD approach that align with what is recommended by Bazzano et al. One such example is: Akama EO, Beres LK, Kulzer JL, et al. A youth- centred approach to improving engagement in HIV services: human- centred design methods and outcomes in a research trial in Kisumu County, Kenya. BMJ Glob Health 2023;8:e012606. doi:10.1136/ bmjgh-2023-012606.  We agree that more detail regarding the final mPROMIS-UE prototype in the results section would strengthen the manuscript, so we have added this.  We agree that “identification” is a strong word choice, so have changed this. | Lines 338-346 edited to state: “This prototype corresponds with “mPROM v1.0” in our published protocol [16]. It provides instructions using text, icons, as well as audio. It asks patients to select among four avatars that are gender neutral but vary in skin tone, in order to represent racial and ethnic diversity. Each question is accompanied by audio and a GIF, and the GIF features the avatar that the patient selected. Each answer response is paired with an illustration, and also has an audio option. The audio, GIFs, and illustrated scale are deployed only if the patient clicks the corresponding icon. A progress bar indicates how many questions have been completed. Unanswered questions are highlighted if the user tries to submit an incomplete form.”  Line 245 edited to state: “Our interviews revealed two distinct user personas:”  Line 249 edited to state: “Our interviews also revealed three literacy personas:” |
| I have a parallel comment for the body of the paper. I recommend reflecting on what is "methods" and what is "results," and how to shift to a more appropriate balance. I imagine that you're hoping to convey to readers the fruit of your effort, rather than the process of developing it. | Please see above comment. We agree that more detail regarding the final mPROMIS-UE prototype in the results section would strengthen the manuscript, so we have added this. | Lines 338-346 edited to state: “This prototype corresponds with “mPROM v1.0” in our published protocol [16]. It provides instructions using text, icons, as well as audio. It asks patients to select among four avatars that are gender neutral but vary in skin tone, in order to represent racial and ethnic diversity. Each question is accompanied by audio and a GIF, and the GIF features the avatar that the patient selected. Each answer response is paired with an illustration, and also has an audio option. The audio, GIFs, and illustrated scale are deployed only if the patient clicks the corresponding icon. A progress bar indicates how many questions have been completed. Unanswered questions are highlighted if the user tries to submit an incomplete form.” |
| In your introduction, you write that your first goal was to create the mPROMIS-UE, and the second goal was to assess its acceptability. Were those the goals of this paper, or the project overall? If the former, check to ensure that the paper's focus is on these objectives. | Thank you for this comment. We agree that these goals were more for the project overall rather than for the paper. We have revised the manuscript to reflect this. | Deleted: “The secondary goal was to confirm the completeness of our findings and the acceptability of the mPROMIS-UE.” |
| Your community advisory board does not have very many members. It may be helpful to contextualize its size with reference to work by others, and to consider listing as a limitation that your user feedback came from a relatively small number of users. | Thank you for this recommendation. Our CAB has 8 members. The recommended number of CAB members is usually between 10-16 (Kubicek, K. and Robles, M. (2016, November 11). Resource for Integrating Community Voices into a Research Study: Community Advisory Board Toolkit), although the published literature reports CABs with as few as 8 members (Lawrence C, Stewart K. The Challenge of Community Representation: Lessons From Six HIV Clinical Research Community Advisory Boards in Uganda. Journal of Empirical Research on Human Research Ethics. 2016;11(4):311-321. doi:10.1177/1556264616665760).  We agree that our community advisory board is on the lower end of what is recommended in terms of size. We agree that adding this as a limitation would strengthen the paper, and have updated the manuscript accordingly. | Added lines 402-404: “Additionally, our community advisory board included only eight members, so our community input beyond those directly associated with the study came from a relatively small number of individuals.” |
| What is the relevance for low-literacy populations outside of the US? Could your tool be employed elsewhere, or just with the population for which it was designed through HCD? | Thank you for this question. This instrument could be employed outside of the US in other English-speaking countries in which the PROMIS Upper Extremity instrument is validated. We have added this point in the implications section of our discussion. | Lines 372-374 edited: “mPROMIS-UE and the MAP provide a novel option for collecting PROs in English-speaking patients of mixed literacy, in the United States and other English-speaking countries in which the PROMIS-UE is validated.” |
