# Supplementary material for: Developing a multimedia patient-reported outcomes measure for low literacy patients with a human-centered design approach
Source: PLoS One. 2024 Jun 5;19(6):e0304351. doi: 10.1371/journal.pone.0304351 (PMC11152264; doi:10.1371/journal.pone.0304351)
Supplement: Attachment — Submitted filename: Response to Reviewers PONE-D-23-24670_R2.doc [file pone.0304351.s003.doc]

**PONE-D-23-24670R1 - Developing a multimedia patient-reported outcomes measure for low literacy patients with a human-centered design approach**

| **Numbered Reviewer Remark and Manuscript Line Number** | **Author Response** | **Revised Manuscript Line Number (in clean manuscript document) and Text Change** |
| --- | --- | --- |
| **Comments to the Author** | | |
| 1. Does the manuscript adhere to the experimental procedures and analyses described in the Registered Report Protocol?  If the manuscript reports any deviations from the planned experimental procedures and analyses, those must be reasonable and adequately justified.  Reviewer #2: Yes  **Reviewer #3: Partly** | This manuscript is intended to represent the findings from the first of four stages of the MAP reported in the Registered Report Protocol. Because this first stage (forward adaptation) includes seven phases, we believe that it is appropriate to report this as a standalone study. We look forward to reporting the findings from the remaining stages of the MAP in subsequent manuscript submissions to PLOS One. | Lines 95-96 edited: “The goal of this study was to demonstrate the feasibility of adapting PROMs to multimedia versions by executing the first stage of the MAP, forward adaptation…” |
| 2. If the manuscript reports exploratory analyses or experimental procedures not outlined in the original Registered Report Protocol, are these reasonable, justified and methodologically sound?  A Registered Report may include valid exploratory analyses not previously outlined in the Registered Report Protocol, as long as they are described as such.  Reviewer #2: Yes  **Reviewer #3: Partly** | By addressing the specific reviewer concerns listed below with substantive edits, we hope we have addressed the reason for Reviewer 3 answering “Partly” to this question. If not, please let us know and we would be happy to consider additional revisions. | See below |
| 3. Are the conclusions supported by the data and do they address the research question presented in the Registered Report Protocol?  The manuscript must describe a technically sound piece of scientific research with data that supports the conclusions. The conclusions must be drawn appropriately based on the research question(s) outlined in the Registered Report Protocol and on the data presented.  Reviewer #2: Yes  Reviewer #3: Yes | Thank you, we agree. | No changes |
| 4. Have the authors made all data underlying the findings in their manuscript fully available?  The PLOS Data policy [plosone.org] requires authors to make all data underlying the findings described in their manuscript fully available without restriction, with rare exception (please refer to the Data Availability Statement in the manuscript PDF file). The data should be provided as part of the manuscript or its supporting information, or deposited to a public repository. For example, in addition to summary statistics, the data points behind means, medians and variance measures should be available. If there are restrictions on publicly sharing data—e.g. participant privacy or use of data from a third party—those must be specified.  Reviewer #2: Yes  Reviewer #3: Yes | Thank you, we agree. | No changes |
| 5. Is the manuscript presented in an intelligible fashion and written in standard English?  PLOS ONE does not copyedit accepted manuscripts, so the language in submitted articles must be clear, correct, and unambiguous. Any typographical or grammatical errors should be corrected at revision, so please note any specific errors here.  Reviewer #2: Yes  Reviewer #3: Yes | Thank you. | No changes |
| 6. Review Comments to the Author  **Reviewer #2:** |  |  |
| Thank you for your edits to strengthen the manuscript. It is much better than your last version. Thank you for your responses to my observations and recommendations. | Thank you. | No changes |
| I appreciate that you changed some instances of persona development to “…our interviews revealed two distinct user personas…” I would call attention to the reality that your abstract still retains the use of “identify,” asserting that you “identified two distinct user personas…” As in my last review, I find this word choice to be overly strong, since another HCD process could conceivably reveal alternative personas. In keeping with the qualitative nature of this undertaking, I wonder if you would please locate continued uses of “identify” in relation to personas and change to a different verb. | Thank you for identifying this oversight. We edited our abstract so that it also reflects the updated language. | Line 42: Edited to, “Our interviews revealed…” |
| **Reviewer #3:** |  |  |
| ABSTRACT  28: The authors may wish to instead refer to their previously published Multimedia Adaptation Protocol. | Thank you, we agree. | Line 29-30: Edited to, “Our study in which we applied the Multimedia Adaptation Protocol included seven phases completed in a serial, iterative fashion…” |
| 53-54: In the abstract, the authors refer to “feasibility of adapting PROMs to multimedia versions”. As I read the manuscript, I understand this to be the primary goal. Please consider clarifying the goal in the manuscript. | Thank you, we agree that the manuscript could be strengthened by a clearer statement of the primary study goal. This has been added to our introduction. | Line 95-98: Added, “The goal of this study was to demonstrate the feasibility of adapting PROMs to multimedia versions by executing the first stage of the MAP, forward adaptation, to adapt the PROMIS-UE to the mPROMIS-UE. This would result in the first multimedia PROM that could be self-administered in a mixed literacy patient population.” |
| 57: Please delete the term “reliably”. The results do not provide any evidence of reliability and reliability is not discussed in the manuscript. | Thank you, we agree. | Line 58: Deleted “reliably” |
| INTRODUCTION  60-61: Given the broad readership, it is important to explain what PROs are. Please provide a definition so readers can understand what kind of outcomes this refers to. | Thank you, we agree. | Line 61-64: Added, “The patient perspective can be captured by patient-reported outcomes (PROs), which are assessments of a health condition made directly by the patient without interpretation or estimation by clinicians or anyone else.”  Added reference:  Health USDo, Human Services FDACfDE, Research, Health USDo, Human Services FDACfBE, Research, et al. Guidance for industry: patient-reported outcome measures: use in medical product development to support labeling claims: draft guidance. Health Qual Life Outcomes. 2006;4:79. |
| 76: “to accurately capture PROs”. Is accuracy the main goal? The study seems to focus more on accessibility, appropriateness, and feasiblity, which are important goals from an equity point of view. Please consider clarifying this here. | Thank you for raising this point. We agree that equitably capturing PROs is a goal of this work, and therefore have edited the manuscript to include this. We do believe that accurately capturing PROs is an additional goal and potential benefit of this work because low literacy patients who have difficulty understanding and completing PROMs may provide responses that are not accurate reflections of their experiences. We have added a sentence in the manuscript to clarify this. | Line 76: Edited, “To capture PROs more equitably…”  Lines 77-78: Added, “This may also allow us to capture PROs more accurately by ensuring that low literacy patients’ responses are accurate reflections of their experiences [12].”  Added reference:  Chang EM, Gillespie EF, Shaverdian N. Truthfulness in patient-reported outcomes: factors affecting patients' responses and impact on data quality. Patient Relat Outcome Meas. 2019;10:171-86. |
| 77: The specific focus on “hand and upper extremity patient population” comes as a surprise, given the flow of the argument in the discussion. Please explain in the introduction why you are focusing on this particular measure and patient population. If the intent is for this to serve as a context for demonstrating the application of the protocol, please say so. Or, else, provide another rationale for this specific focus.  Additionally, given the broad readership of this journal, please describe further in the introduction what is meant by "hand and upper extremity patients" and provide some description of how PROMs are being used in this context (e.g., To determine the best treatment options? Or to evaluate the outcomes of surgical or medical interventions?). | Thank you for noting this. We agree and have added an additional paragraph to address the important points that the reviewer brings up. | Lines 84-94: Added, “To execute and demonstrate the application of the MAP, we elected to adapt the Patient Reported Outcomes Measurement Information System Upper Extremity (PROMIS-UE) to a multimedia version (mPROMIS-UE) in a hand and upper extremity surgery patient population. In the U.S., an estimated 3.5 million patients sustain hand and upper extremity trauma each year [14], with more than 1.1 million upper extremity ambulatory procedures performed annually [15]. In hand and upper extremity care, growing evidence of the divergence between patient and provider perception of health and functional status has driven an increase in the use of PROs as outcome measures in research and to guide patient-centered and value-based clinical care [16-19]. The PROMIS-UE and the hand and upper extremity population are ideal for the first application of the MAP because variation in physical function, such as in upper extremity function, may be easier to convey in multimedia elements than variations in mental or social health.”  Added references:  - Ootes D, Lambers KT, Ring DC. The epidemiology of upper extremity injuries presenting to the emergency department in the United States. Hand (New York, NY). 2012;7(1):18-22.  - Jain NB, Higgins LD, Losina E, Collins J, Blazar PE, Katz JN. Epidemiology of musculoskeletal upper extremity ambulatory surgery in the United States. BMC Musculoskelet Disord. 2014;15:4. |
| 81: “MAP is an innovative protocol for adaptation”. This reads rather repetitive, stating the obvious. Please consider revising. | Thank you, we agree. | Line 79-81: Edited, “…we developed the Multimedia Adaptation Protocol (MAP) [13], which adapts validated, text-based PROMs to a multimedia format…” |
| METHODS  Overall comment: Please refer to the journal's guidelines on reporting of qualitative research. A few key points are identified in my comments above.  [https://journals.plos.org/plosone/s/submission-guidelines#loc-guidelines-for-specific-study-types%20[journals.plos.org](https://journals.plos.org/plosone/s/submission-guidelines" \l "loc-guidelines-for-specific-study-types [journals.plos.org)]  "Qualitative research studies should be reported in accordance to the Consolidated criteria for reporting qualitative research (COREQ) checklist or Standards for reporting qualitative research (SRQR) checklist." | Thank you, we agree. We have reviewed the SRQR and have edited the manuscript to ensure that it follows these best practice guidelines. Some of these changes are reflected in the manuscript changes described elsewhere in this document.  We also reported our findings in accordance with what is recommended for health research involving design, so have clarified this and added the relevant reference. | Title edited to, “Developing a multimedia patient-reported outcomes measure for low literacy patients with a human-centered design approach”  Edited lines 145-147: “This study… is reported according to the Standards for Reporting Qualitative Research (SRQR) [20] as well as Bazzano et al.’s reporting recommendations for health research involving design [41].”  Added reference:  Bazzano AN, Yan SD, Martin J, Mulhern E, Brown E, LaFond A, et al. Improving the reporting of health research involving design: a proposed guideline. BMJ Glob Health. 2020;5(2):e002248. |
| Study setting (101): To address my comment above, please explain how PRO data are currently being used in this clinic. What are the purposes for collecting the PRO data? | Thank you, we agree that this would strengthen the manuscript. | Line 114-115: Added, “These PROs are used for research and for direct clinical care including patient education and progress tracking.” |
| Instrument selection (113): Please provide further description of the items and measurement validity evidence. Given the focus of this manuscript, it is important to fully understand what is being measured and what the validity evidence of the original instrument is. | Thank you, we agree that this would strengthen the manuscript. | Line 126-132: Added, “It has demonstrated sufficient construct validity with moderate to strong correlation with the legacy instrument Quick Disabilities of Arm, Shoulder, and Hand (QuickDASH) (*r* = -0.47 to -0.83) [25, 26], good internal consistency (Cronbach alpha coefficient ranging between 0.78-0.93) [27-30], and good responsiveness (AUC 0.81-0.82) [29, 30]. Regarding content validity, PROMIS-UE has not been found to be limited by floor effects, but there are mixed findings for ceiling effects [29, 31-36].”  Added references:  - Jayakumar P, Teunis T, Vranceanu AM, Lamb S, Williams M, Ring D, et al. Construct Validity and Precision of Different Patient-reported Outcome Measures During Recovery After Upper Extremity Fractures. Clin Orthop Relat Res. 2019;477(11):2521-30.  - Doring AC, Nota SP, Hageman MG, Ring DC. Measurement of upper extremity disability using the Patient-Reported Outcomes Measurement Information System. The Journal of hand surgery. 2014;39(6):1160-5.  - Speck RM, Ye X, Bernthal NM, Gelhorn HL. Psychometric properties of a custom Patient-Reported Outcomes Measurement Information System (PROMIS) physical function short form and worst stiffness numeric rating scale in tenosynovial giant cell tumors. J Patient Rep Outcomes. 2020;4(1):61.  - England DL, Miller TA, Stevens PM, Campbell JH, Wurdeman SR. Assessment of a 9-item PROMIS Upper Extremity Instrument among Individuals with Upper Limb Amputation. American journal of physical medicine & rehabilitation / Association of Academic Physiatrists. 2020.  - Yoon AP, Kaur S, Chou CH, Chung KC, Malay S, Shauver M, et al. Reliability and Validity of Upper Extremity Patient-Reported Outcome Measures in Assessing Traumatic Finger Amputation Management. Plast Reconstr Surg. 2020;145(1):94e-105e.  - Kaat AJ, Rothrock NE, Vrahas MS, O'Toole RV, Buono SK, Zerhusen T, Jr., et al. Longitudinal Validation of the PROMIS Physical Function Item Bank in Upper Extremity Trauma. J Orthop Trauma. 2017;31(10):e321-e6.  - Gausden EB, Levack AE, Sin DN, Nwachukwu BU, Fabricant PD, Nellestein AM, et al. Validating the Patient Reported Outcomes Measurement Information System (PROMIS) computerized adaptive tests for upper extremity fracture care. J Shoulder Elbow Surg. 2018;27(7):1191-7.  - Iyer S, Koltsov JCB, Steinhaus M, Ross T, Stein D, Yang J, et al. A Prospective, Psychometric Validation of National Institutes of Health Patient-Reported Outcomes Measurement Information System Physical Function, Pain Interference, and Upper Extremity Computer Adaptive Testing in Cervical Spine Patients: Successes and Key Limitations. Spine (Phila Pa 1976). 2019;44(22):1539-49.  - Rojas EO, Glass N, Owens J, Anthony CA, Bollier M, Wolf BR, et al. Performance of the PROMIS in Patients Undergoing 3 Common Elbow Procedures. Orthop J Sports Med. 2019;7(6):2325967119852595.  - Hajewski CJ, Glass NA, Westermann RW, Bollier M, Wolf BR, Hettrich C. Performance of the PROMIS After Operative Interventions for Shoulder Instability. Orthop J Sports Med. 2019;7(5):2325967119846920.  - Anthony CA, Glass N, Hancock K, Bollier M, Hettrich CM, Wolf BR. Preoperative Performance of the Patient-Reported Outcomes Measurement Information System in Patients With Rotator Cuff Pathology. Arthroscopy. 2017;33(10):1770-4 e1. |
| Study design  141: Please provide a reference to participant observation methods. | Thank you, we have done this. | Added reference:  Mack N, Woodsong C, MacQueen K, Guest G, Namey E. Qualitative Research Methods: A Data Collector's Field Guide: Family Health International; 2005. |
| 149: Please define what this means. How is this different from regular thematic analysis? The description of the results reflect a more general content analysis approach. Please describe how the data were analyzed to arrive at themes. Or, if content analysis was used, please indicate this. Either way, please provide a suitable reference for the approach that was used. | Thank you for this prompt to clarify our analysis approach; we agree that more details regarding our methodological and analytic approach are needed.  Our methods are grounded in qualitative analysis as applied to design thinking. There are several differences between a traditional qualitative paradigm that uses a “regular thematic analysis” and the HCD paradigm that we adopted. One difference is in how findings are synthesized. In the former, researchers typically develop a codebook and use memos, matrices, and models. In the latter: “The process of arriving at insights from fieldwork is more of a creative than scientific process, with less emphasis on creating an audit trail that can be replicated by others. Affinity mapping is one key strategy for synthesizing HCD findings. It involves a process of looking for key themes across different types of data, visually organizing these themes and supporting observations (oftentimes using color-coded sticky notes) and then seeking to identify insights – in the form of user needs or preferences, ways to overcome barriers or encourage behavior change” (Tolley 2017).  We have clarified our analysis approach and added the relevant references. | Lines 168-173: Added, “Data were qualitatively analyzed using design-oriented, rapid thematic analysis including iterative reading, insight identification using inductive and abductive thinking, and affinity clustering. Resulting clusters and early insights were reviewed by a second team member (LKB) to solidify themes; these themes became the deductive themes used in the subsequent analysis of discovery interviews.”  Line 210-211: Added, “Our rapid thematic analysis aligns with the study’s HCD approach.”  Added references:  - Vindrola-Padros C, Johnson GA. Rapid Techniques in Qualitative Research: A Critical Review of the Literature. Qual Health Res. 2020;30(10):1596-604.  - Lewinski AA, Crowley MJ, Miller C, Bosworth HB, Jackson GL, Steinhauser K, et al. Applied Rapid Qualitative Analysis to Develop a Contextually Appropriate Intervention and Increase the Likelihood of Uptake. Medical care. 2021;59(Suppl 3):S242-S51.  -Tolley B. Traditional Socio-Behavioral Research And Human-Centered Design: Similarities, Unique Contributions and Synergies. FHI 3602017 [Available from: <https://theimpt.org/traditional-socio-behavioral-research-and-human-centered-design-similarities-unique-contributions-and-synergies/>.  -Dam RF, Siang TY. Affinity Diagrams: How to Cluster Your Ideas and Reveal Insights. Interaction Design Foundation - IxDF2022 [Available from: https://www.interaction-design.org/literature/article/affinity-diagrams-learn-how-to-cluster-and-bundle-ideas-and-facts.  -Kolko J. Abductive Thinking and Sensemaking: The Drivers of Design Synthesis. Design Issues. 2010;26(1). |
| Discovery interviews: Some inclusion criteria are described, but the sampling approach is unclear. Please describe how participants were selected and the type of sampling method used (e.g., purposive, convenience, consecutive, snowball). Did any of the approached people refuse to participate? If so, is anything known about the reasons for non-participation? | Thank you, we have added these details to the methods. | Lines 182-186: Added, “Participants were screened for eligibility through standard clinic in-take forms. Those who met inclusion criteria were approached for participation using a purposive sampling strategy [49] developed to ensure heterogeneity of literacy levels in our cohort. Specifically, we used education as a proxy for literacy and only recruited patients who reported an education level of high school or less.”  Lines 187-188: Added, “Reasons for non-participation and characteristics of the non-participant cohort were not recorded.”  Added reference:  Martinez-Mesa J, Gonzalez-Chica DA, Duquia RP, Bonamigo RR, Bastos JL. Sampling: how to select participants in my research study? An Bras Dermatol. 2016;91(3):326-30. |
| 171: Please indicate here that the CLA was the interviewer and describe the nature of the relationship of CLA with the interview participants. Is it possible that he results may have been influenced by the relationship. | Thank you for this recommendation, we have done this. To support reflexivity during analysis, intermediate analyses were conducted with a second team member (LKB); this has also been clarified in the manuscript. | Lines 197-198: Added, “…by CLA, a researcher with no clinical relationship with patients and no employment relationship with clinic staff…”  Lines 204-205: Added, “Subsequent analyses were conducted jointly by two research team members (CLA and LKB) to support reflexivity during analysis.” |
| 172: Please provide further description of the interview guides (e.g., provide examples of questions asked or include the guides as an appendix). | Thank you for this recommendation. We have attached the three discovery interview guides tailored to each participant group as an appendix. | Line 200: Added, “S1. Appendix”  Line 473: Added, “S1 Appendix. Discovery interview guides for patients, caregivers, and clinic staff” |
| 178: Was CLA the only person doing the analyses, or were others involved in the analysis as well? Please describe. | Thank you for this question. CLA performed the preliminary analysis (writing analytic memos), but all subsequent analyses were conducted jointly by CLA and LKB. We have added a sentence to clarify this. | Line 204-205: Added, “Subsequent analyses were conducted jointly by two research team members (CLA and LKB) to support reflexivity during analysis.” |
| 179: Please provide a reference to the thematic analysis approach that was used (given that there are different methodological approaches - see above comment). Please also indicate whether any software was used to assist with the analysis. | Thank you; we have done as recommended. Please also see comments above regarding our analysis approach. | Line 207-212: Added, “We conducted rapid framework-guided thematic analysis [43-45, 56] using the deductive themes identified during observation and identified additional themes inductively via affinity clustering and categorization. […] Our rapid thematic analysis aligns with the study’s HCD approach. Microsoft Word and Excel software (Redmond, WA) were used to conduct analyses.”  References added:  - Vindrola-Padros C, Johnson GA. Rapid Techniques in Qualitative Research: A Critical Review of the Literature. Qual Health Res. 2020;30(10):1596-604.  - Lewinski AA, Crowley MJ, Miller C, Bosworth HB, Jackson GL, Steinhauser K, et al. Applied Rapid Qualitative Analysis to Develop a Contextually Appropriate Intervention and Increase the Likelihood of Uptake. Medical care. 2021;59(Suppl 3):S242-S51  - Gale RC, Wu J, Erhardt T, Bounthavong M, Reardon CM, Damschroder LJ, et al. Comparison of rapid vs in-depth qualitative analytic methods from a process evaluation of academic detailing in the Veterans Health Administration. Implement Sci. 2019;14(1):11.  - Nevedal AL, Reardon CM, Opra Widerquist MA, Jackson GL, Cutrona SL, White BS, et al. Rapid versus traditional qualitative analysis using the Consolidated Framework for Implementation Research (CFIR). Implement Sci. 2021;16(1):67. |
| Ideation workshop: It seems that patients were not included in this phase. Is that correct? Please make this explicit, as this could be viewed as a limitation. | Thank you for raising this point. Patients were not included; this has been made explicit. | Line 219-221: Added, “Although patients did not participate in the ideation workshop, we engaged co-creation and participatory processes [58, 59] by incorporating direct patient quotes and patient suggestions into the discussion.”  Added references: -Crosby S, Younie S, Williamson I, Laird K. Evaluating approaches to designing effective Co-Created hand-hygiene interventions for children in India, Sierra Leone and the UK. PLoS One. 2020;15(9):e0239234.  -Wolstenholme D, Kidd L, Swift A. Co-creation and co-production in health service delivery: what is it and what impact can it have? Evid Based Nurs. 2019;22(4):97-100. |
| RESULTS  Direct observation: See comment above regarding content vs. thematic analysis. The results, as presented here, seem to be more representative of a type of content analysis. It is unclear what the inductive themes might be. Please revise or clarify. | Thank you. Please see our comments above regarding our analytic approach. To further clarify, we did not use traditional content analysis, but rather used a rapid thematic analysis that aligns with our HCD approach. Please see the manuscript edits and added references to support our methods. | Lines 168-173 edited to clarify the methods used during analysis of observation data: “Data were qualitatively analyzed using design-oriented, rapid thematic analysis [43-45] including iterative reading, insight identification using inductive and abductive thinking, and affinity clustering [46-48]. Resulting clusters and early insights were reviewed by a second team member (LKB) to solidify inductive themes; these themes became the deductive themes used in the subsequent analysis of discovery interviews.”  Line 210-211: Added, “Our rapid thematic analysis aligns with the study’s HCD approach.”  Added references:  - Vindrola-Padros C, Johnson GA. Rapid Techniques in Qualitative Research: A Critical Review of the Literature. Qual Health Res. 2020;30(10):1596-604.  - Lewinski AA, Crowley MJ, Miller C, Bosworth HB, Jackson GL, Steinhauser K, et al. Applied Rapid Qualitative Analysis to Develop a Contextually Appropriate Intervention and Increase the Likelihood of Uptake. Medical care. 2021;59(Suppl 3):S242-S51  - Gale RC, Wu J, Erhardt T, Bounthavong M, Reardon CM, Damschroder LJ, et al. Comparison of rapid vs in-depth qualitative analytic methods from a process evaluation of academic detailing in the Veterans Health Administration. Implement Sci. 2019;14(1):11.  - Nevedal AL, Reardon CM, Opra Widerquist MA, Jackson GL, Cutrona SL, White BS, et al. Rapid versus traditional qualitative analysis using the Consolidated Framework for Implementation Research (CFIR). Implement Sci. 2021;16(1):67. |
| Ideation workshop: It is unclear what all the idea names in Table 3 refer to. Please provide further description in the text or a footnote to this table. | Thank you, we agree that this needed additional explanation and have added this. | Line 337-338: Added, “Each idea for addressing the design challenge was given a specific name to facilitate identification and discussion of the various ideas (final column, Table 3).” |
| Prototyping: Based on the MAP approach, I understand that the prototype should be informed by the results of the prior phases (B, C and D). However, this is currently not clear. Please expand the text, or in the table, to more explicitly demonstrate how the prototype was developed based on the results reported above. | Thank you, we agree that adding this would strengthen the manuscript, so we have added an additional column in Table 4 to explicitly report this. | Line 351-353: Edited, “Table 4 details which design challenge(s) each prototype feature was designed to address, how it did so, and examples of how these decisions were informed by findings from prior phases.”  Line 355: An additional column added to Table 4, titled “How Decisions were Informed by Findings from Prior Phases.” This column includes:   - Literacy: “The availability of audio, GIFs, and the illustrated response scale addresses the needs and preferences of the ‘illiterate’ and ‘slow reader’ literacy personas that our discovery interviews revealed. Their availability is important even for patients who have caregivers, which was also identified during discovery interviews.” - Customizability: “The ability to customize the experience allows high literacy users who do not need the multimedia features to avoid them, potentially shortening the time of survey completion. This was guided by the ‘survey fatigue’ and ‘timing’ themes identified during direct observation and ‘length and timing’ and ‘customizability’ themes from discovery interviews. This also caters to the needs and preferences of all the distinct user and literacy personas that our discovery interviews revealed.” - Convenience: “Convenience afforded by the smartphone platform was guided by the ‘setting’ and ‘role of technology’ themes from direct observation, and the ‘location’ and ‘platforms and technology’ themes from discovery interviews.   - “Convenience afforded by the progress indicator provides the ‘do-it-right’ persona the information to decide when and where is best to complete the mPROMIS-UE. The importance of this was guided by the ‘setting’ theme from direct observations.” - Avoiding discomfort: “Inclusion of avatars was guided by the ‘patient-centered goals of PROMs’ theme identified in direct observation and ‘anxiety and shame’ theme that emerged from discovery interviews.   - Customizability gives patients the ability to consider how their setting influences their preferences for using or not using multimedia features to avoid discomfort, which we prioritized due to the ‘setting’ theme from direct observations and ‘anxiety and shame’ theme from discovery interviews. |
| DISCUSSION  It would be helpful, and conventional, to first describe a summary of the overall result of your study | Thank you, we agree and have added this. | Lines 387-396: Added as the first paragraph of our discussion, “By executing forward adaptation, the first stage of the MAP, we successfully adapted the PROMIS-UE to the mPROMIS-UE by addressing the challenges identified by a mixed literacy hand and upper extremity patient cohort. The mPROMIS-UE is customizable and responsive to our five user and literacy personas, convenient in terms of timing, location, and platform, and is designed to avoid potential shame/discomfort. When we shared our initial findings and prototype ideas with patients, caregivers, and our CAB, their responses indicated that our research goals and findings broadly resonated with them. They offered recommendations for improving the mPROMIS-UE in various domains that were integrated into the final mPROMIS-UE prototype. This work demonstrates the feasibility of adapting PROMs to multimedia versions that can be self-administered by mixed literacy populations.” |
| 365: Please discuss what you consider to be the key recommendations for advancing this approach to PRO data capture. | Thank you, we agree that this would strengthen the manuscript, and have added this. | Lines 411-416: Added, “Once these stages are completed, to advance a multimedia approach to capturing PRO data, we recommend future research efforts focus on dissemination, implementation, and expansion. This can include but is not limited to making mPROMIS-UE an official PROMIS instrument that is publicly available for widespread use, deploying it in hand and upper extremity clinics, integrating with the electronic health record, and collaborating with investigators from other specialties to adapt additional PROMs to mPROMs.” |
| It is critically important to indicate the need for further research on validity evidence. The authors correctly refer to this in the abstract, when they state: "“Future research will include back adaptation, usability testing via qualitative evaluation, and psychometric validation of the mPROMIS-UE”. Please elaborate on this in the discussion section of the manuscript. | Thank you, we agree that this would strengthen the manuscript, and have added this. | Lines 404-411 Added, “It however is a prototype and will not be ready for implementation until the remaining stages of the MAP (back adaptation, qualitative evaluation, psychometric validation) [13] have been executed. Back adaptation will ensure that we have not altered the content of the PROMIS-UE during forward adaptation. Qualitative evaluation will ensure a well-designed user interface that increases the likelihood of successful implementation and long-term adoption and minimizes the risk of human error and operating inefficiencies. Psychometric validation is needed to confirm whether the mPROMIS-UE measures the construct it is intended to measure, in the intended population.” |
| 7. PLOS authors have the option to publish the peer review history of their article ([what does this mean? [journals.plos.org]](https://urldefense.com/v3/__https:/journals.plos.org/plosone/s/editorial-and-peer-review-process*loc-peer-review-history__;Iw!!D7IIWT94AA!909QL53Cs8MWiOOy9-C7VUI5XYfdRFjBnQpO0nCVmXdKtE8pIKGcgYhLAmdjHi3c_dbg_CgnjdI04miipCm8I7Sh$)). If published, this will include your full peer review and any attached files.  If you choose “no”, your identity will remain anonymous but your review may still be made public.  **Do you want your identity to be public for this peer review?** For information about this choice, including consent withdrawal, please see our [Privacy Policy [plos.org]](https://urldefense.com/v3/__https:/www.plos.org/privacy-policy__;!!D7IIWT94AA!909QL53Cs8MWiOOy9-C7VUI5XYfdRFjBnQpO0nCVmXdKtE8pIKGcgYhLAmdjHi3c_dbg_CgnjdI04miipJQqi7-B$).  Reviewer #2: No  Reviewer #3: No |  | No changes |
