# Supplementary material for: Developing a multimedia patient-reported outcomes measure for low literacy patients with a human-centered design approach
Source: PLoS One. 2024 Jun 5;19(6):e0304351. doi: 10.1371/journal.pone.0304351 (PMC11152264; doi:10.1371/journal.pone.0304351)
Supplement: Attachment — Submitted filename: Response to Reviewers PONE-D-23-24670R2.doc [file pone.0304351.s004.doc]

**PONE-D-23-24670R2 - Developing a multimedia patient-reported outcomes measure for low literacy patients with a human-centered design approach**

| **Numbered Reviewer Remark and Manuscript Line Number** | **Author Response** | **Revised Manuscript Line Number (in clean manuscript document) and Text Change** |
| --- | --- | --- |
| **Comments to the Author** | | |
| The first mention of PROMIS needs to include a trademark, PROMIS® | Thank you, this has been done. | Line 85: Added “PROMIS®” |
| PROMIS UE is not disease-specific. It is a generic measure that can be used across all diseases and conditions. Please revise accordingly in the "Instrument selection" section. | Thank you, we agree and have updated the manuscript accordingly. | Lines 125-126: Edited to, “The PROMIS-UE is a generic measure that can be used across all diseases and conditions.” |
| In the "Implications" section, the authors stated that "as the mPROMIS-UE could replace the current PROMIS instrument rather than being an additional instrument to be administered to certain segments of the clinic population." This statement was not supported by the evidence provided in this paper, as no thorough psychometric analysis has been conducted for mPROMIS-UE. | Thank you, we agree and have deleted this sentence from the manuscript. | Deleted: “, as the mPROMIS-UE could replace the current PROMIS instrument rather than being an additional instrument to be administered to certain segments of the clinic population.” |
